# Endophytic Fungus Drives Nodulation and N_2_ Fixation Attributable to Specific Root Exudates

**DOI:** 10.1128/mBio.00728-19

**Published:** 2019-07-16

**Authors:** Xing-Guang Xie, Feng-Min Zhang, Teng Yang, Yan Chen, Xiao-Gang Li, Chuan-Chao Dai

**Affiliations:** a Jiangsu Key Laboratory for Microbes and Functional Genomics, Jiangsu Engineering and Technology Research Center for Industrialization of Microbial Resources, College of Life Sciences, Nanjing Normal University, Nanjing, China; b Co-Innovation Center for Sustainable Forestry in Southern China, College of Biology and the Environment, Nanjing Forestry University, Nanjing, China; c National Key Laboratory of Plant Molecular Genetics, Chinese Academy of Sciences Center for Excellence in Molecular Plant Sciences, Institute of Plant Physiology and Ecology, Shanghai Institutes for Biological Sciences, Chinese Academy of Sciences, Shanghai, China; d State Key Laboratory of Soil and Sustainable Agriculture, Institute of Soil Science, Chinese Academy of Sciences, Nanjing, China; University of Hawaii at Manoa; University of Hawaii at Manoa

**Keywords:** *Arachis hypogaea* L., *Bradyrhizobium*, endophytic fungus *Phomopsis liquidambaris* (*Phomopsis*/*Diaporthe*), nodulation and N_2_ fixation, phenolic and flavonoid compounds, root exudates

## Abstract

Endophytic fungi play important roles in the modification of ecosystem productivity; however, the underlying mechanisms are only partly understood. A 2-year field plot experiment verified that the endophytic fungus Phomopsis liquidambaris increased peanut (*Arachis hypogaea* L.) yields and significantly increased nodulation and N_2_ fixation regardless of whether N fertilizers were added. Root exudates collected from P. liquidambaris-colonized plants significantly improved nodulation and N_2_ fixation. Rhizosphere stimulation experiments further showed that colonized root exudates had significantly decreased soil nitrate (NO_3_^−^) concentrations, with decreased abundance and diversity of ammonia oxidizing archaea (AOA). In contrast, the abundance and diversity of diazotrophs significantly increased, and most diazotrophs identified were peanut nodulation-related strains (*Bradyrhizobium* sp.). P. liquidambaris symbiosis increased the expression of phenolic and flavonoid synthesis-related genes, and the derived phenolics and flavonoids could effectively increase the chemotaxis, biofilm formation, and *nodC* gene expression (nodulation-related biological processes) of the *Bradyrhizobium* strain. Metabolic pattern analysis showed that phenolics and flavonoids are more likely to accumulate to higher levels in the rhizosphere soil of peanuts colonized with P. liquidambaris. Finally, a synthetic root exudate experiment further confirmed the underlying mechanisms for the P. liquidambaris-induced improvement in nodulation and N_2_ fixation, i.e., that the specific root exudates derived from P. liquidambaris colonization decrease nitrate concentration and increase the population and biological activities of peanut nodulation-related *Bradyrhizobium* species, which beneficially enhance peanut-*Bradyrhizobium* interactions. Therefore, this study is the ﬁrst to provide new insight into a positive relationship between an exotic endophytic fungus, crop nodulation, and N_2_ fixation increase.

## Abstract (precis)

Endophytic fungi play an important role in balancing the ecosystem and boosting host growth; however, the underpinning mechanisms remain poorly understood. Here, we found that endophytic fungal colonization with P. liquidambaris significantly increased the productivity, nodulation, and N_2_ fixation of peanuts through the secretion of specific root exudates. We provide a reasonable mechanism explaining how P. liquidambaris promotes peanut nodulation and N_2_ fixation, whereby the specific root exudates produced by P. liquidambaris colonization decrease rhizosphere soil nitrate (NO3^−^) and increase the population and biological activities of peanut-nodulating-related *Bradyrhizobium* strains, which is beneficial to enhancing the peanut-*Bradyrhizobium* symbiotic interaction. Our study provides reliable empirical evidence to show the mechanism of how an exotic endophytic fungus drives an increase in nodulation and N_2_ fixation, which will be helpful in erecting a resource-efficient and sustainable agricultural system.

## INTRODUCTION

Endophytic fungi are microorganisms that asymptomatically grow within healthy plant tissues without causing any immediate overt negative effects (1). Many ecosystems, including wetlands, grasslands, forests, and even crop systems, show a positive relationship between endophytic fungal diversity and ecosystem productivity (2–4). Several biological mechanisms have been proposed to explain this relationship. Endophytic fungi themselves facilitate effects, as various fungal metabolites (e.g., phytohormone analogues, alkaloids, and volatiles) are helpful in promoting plant growth and enhancing host resistance to pathogen infections, insects, or herbivore feeding (3, 5, 6). Plant physiology-facilitative effects (PPFE) result when endophytic fungal colonization leads to the enhancement of host plant physiology, enabling plants to counter various biotic and abiotic stresses (7, 8). Plant rhizosphere facilitative effects (PRFE) include the stimulation of longer root hairs and enhanced exudation of secondary metabolites (phenolic-like compounds) into the rhizosphere induced by endophytic fungi, resulting in more efficient absorption of soil nutrients and plant-microbe interactions (3, 9).

Biological nitrogen fixation has been widely studied in the functioning of ecosystems because it is regarded as one of the most important processes involved in ecosystem access to available N (10, 11). Leguminous peanuts (Papilionoideae, *Arachis hypogaea* L.), one of the most important oil and cash crops cultivated in over 100 countries, can use N_2_ through symbiosis with the soil *Bradyrhizobium* genus to form a new organ, the root nodule (12). However, long-term continuous cropping has seriously affected peanut yield and quality (13, 14), and the decreases in nodulation and N_2_ fixation are considered important factors contributing to the obstacles in peanut replanting (15, 16). The genus *Phomopsis* is one of the most diverse and ecologically important groups of microfungi and has been reported as pathogens, endophytes, and saprobes existing in natural ecosystems (17). As a large and improvable microbial resource, some species of *Phomopsis* have been considered to play important roles in the effective control of plant diseases and host growth (17, 18). In our previous study, a broad-spectrum endophytic fungus, Phomopsis liquidambaris, belonging to *Sphaeropsidaceae* of *Sphaeropsidales* in *Coelomycetes*, was isolated from the inner bark of the stem of Bischoﬁa polycarpa (Euphorbiaceae) (19). As an environmentally adaptive fungus, P. liquidambaris can efﬁciently degrade various toxic compounds and regulate soil activity and microbial community structure (9, 20–22). As a symbiotic fungus, P. liquidambaris was observed to signiﬁcantly promote the growth of rice and peanut by establishing a symbiotic relationship (16, 18, 23, 24). Even more interesting, our further study found that root colonization with the endophytic fungus P. liquidambaris (here, P treatment) could significantly increase peanut nodulation and N_2_ fixation (16, 24, 25). It is well known that the establishment of a reciprocal symbiotic process between legumes and the rhizobium involves complex molecular dialogues (12, 26), so the optimal nodulation physiology of both the host and the rhizobium is crucial for forming this symbiotic interaction. Our recent studies demonstrated that P. liquidambaris colonization improved peanut PPFE, including increasing plant carbohydrate metabolism, enhancing H_2_O_2_, NO, and auxin symbiotic signaling responses, and increasing lateral root formation (16, 24, 25, 27). To date, the mechanisms of nodulation increase produced by PPFE have been extensively studied, but less attention has been directed to the effects of hosts colonized with P. liquidambaris on the peanut PRFE. In fact, many studies have also suggested that the appropriate rhizosphere microecological environment is responsible for increasing legume-rhizobium symbiotic interaction (28, 29).

Plant root exudates contain various organic and inorganic components that play crucial roles in regulating PRFE, including rhizosphere soil nutrient cycling, microbial community structure, and signal transduction between the root system and the soil interface (13, 30, 31). Root exudates affect soil nutrients, especially high concentrations of available N, and it has been suggested that there are multiple inhibition effects on nodule formation and development (10, 32, 33). Root exudates affect N transformation-related microorganisms, such as ammonia-oxidizing bacteria and archaea (AOB and AOA, respectively) and diazotrophs, and were found to be closely related to rhizosphere N availability (34–36). Additionally, the number and species of N_2_-fixing rhizobia in soil are directly responsible for nodulation and N_2_ fixation abilities (12, 37). Root exudates also affect symbiosis signal transduction, especially for phenolic and flavonoid compounds, which are important signaling molecules in regulating nodulation-related biological activities of rhizobia (11, 12, 38). Therefore, we hypothesized that the secretion of specific root exudates caused by P. liquidambaris colonization and their effects on PRFE are important for increasing nodulation and N_2_ fixation.

Many studies have demonstrated that the quantity and quality of root exudates are determined by plant species and also significantly depend on the degree of symbiosis of exogenous microorganisms (31, 39, 40). In our previous study, we found that colonization by the endophytic fungus P. liquidambaris could alter the secretion of organic compounds by rice roots and increase the concentration of soluble saccharides, total free amino acids, and organic acids in root exudates (23). These effects were found to play an important role in reducing fertilizer input and increasing rice productivity in N-limited soils. In addition, further studies have demonstrated that P. liquidambaris symbiosis increases the secretion of ﬂavonoid compounds in peanut root exudates (24). However, whether the specific root exudates caused by P. liquidambaris colonization can increase peanut nodulation and N_2_ fixation by improving PRFE is still unclear. Therefore, this study is aimed at determining the effects of root exudate changes caused by P. liquidambaris colonization on PRFE, with the goal of proposing a new mechanism to explain how endophytic fungi increase crop nodulation and N_2_ fixation capacities.

## RESULTS

### Evaluation of peanut nodulation characteristics in field plots.

**FIG 1:**
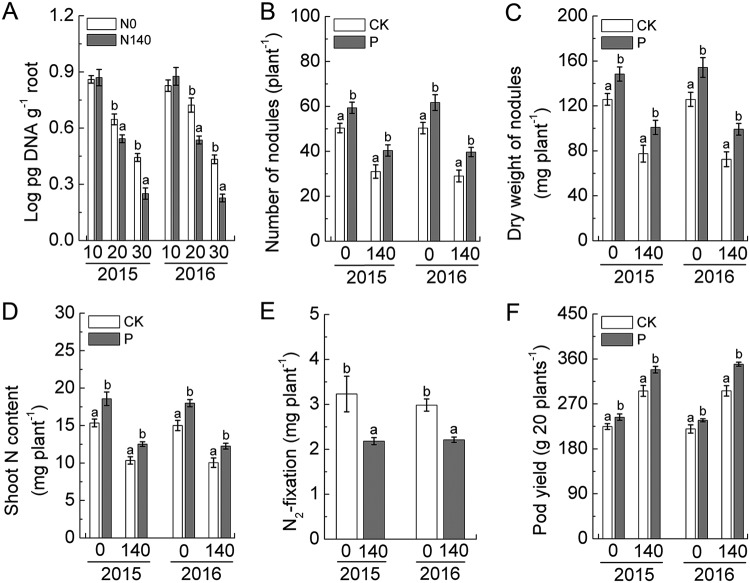
(A) qPCR detection of P. liquidambaris colonization in peanut roots after 10, 20, and 30 days of planting under field plot conditions. Effects of P. liquidambaris colonization on peanut nodule number (B), dry weight of nodules (C), and N_2_ fixation (D and E) after 60 days of planting are shown. (F) Effects of *P. liquidambaris* colonization on peanut pod yield after harvest. Error bars represent means ± standard deviations of three biological replicates, with each biological replicate representing a pooled sample from at least six individual plants. The data in columns marked by different lowercase letters are significantly different between the different treatments, and the same letters or no letters indicate no significant difference. N application rates were 0 and 140 kg ha^−1^.

A consecutive 2-year field plot experiment found that low soil N availability (no N fertilizer application) could significantly increase peanut nodulation and N_2_ fixation capacities compared with levels when 140 kg ha^−1^ N fertilizer was applied (Fig. 1B to D). Further analysis showed that soil N availability was also responsible for affecting the amount of P. liquidambaris colonization in peanut roots, with P. liquidambaris colonization significantly increased in plots without added N fertilizer compared to levels in those plots fertilized with 140 kg ha^−1^ N (Fig. 1A). P. liquidambaris colonization (P treatment) produced a higher number of peanut nodules than noncolonization (CK treatment, treated with sterile distilled water) and increased nodule dry weights and shoot N content even under high-N fertilization conditions (Fig. 1B to D). Equally interesting is that the colonization of P. liquidambaris resulted in an enhancement of peanut N_2_ fixation regardless of whether N fertilizer was added (Fig. 1E). In addition, P. liquidambaris colonization increased peanut pod yield compared to that of the CK-treated plants (Fig. 1F).

### Effects of root exudates on peanut nodulation.

**FIG 2:**
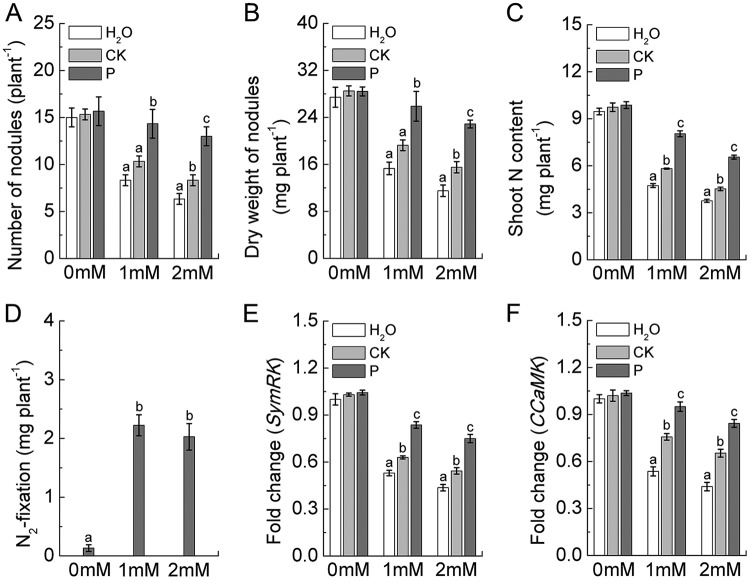
Effects of root exudates and N supplies on peanut nodule number (A), dry weight of nodules (B), N_2_ fixation (C and D), and symbiosis-related gene expression (*SymRK* and *CCaMK*) (E and F) are shown. Data are means ± standard deviations of three biological replicates, with each biological replicate representing a pooled sample from at least six individual plants. The data in columns marked by different lowercase letters are significantly different between the different treatments, and the same letters or no letters indicate no significant difference. Concentrations of nitrate (NO_3_^−^) applied are indicated.

When 1 mM nitrate (NO_3_^−^) was added to the experimental medium, P. liquidambaris-colonized root exudates (P treatment) significantly increased the numbers of nodules on peanut roots and the dry weight of nodules and peanut shoot N content compared to levels with the CK (noncolonized root exudates) and H_2_O treatments (Fig. 2A to C). In this study, although the high concentration of nitrate (2 mM) had a slight inhibitory effect on peanut nodulation traits, when nitrate application was increased to 2 mM, the peanut roots treated with P. liquidambaris-colonized root exudates still retained relatively higher nodulation characteristics than those with the CK treatment. Additionally, compared to levels with the CK treatment, the addition of P. liquidambaris-colonized root exudates led to a significant increase in peanut N_2_ fixation capacity (Fig. 2D). In addition, further analysis showed that the expression levels of peanut-*Bradyrhizobium* symbiosis-related genes (*SymRK* and *CCaMK*) in peanut roots were also significantly enhanced when peanut roots were treated with P. liquidambaris -colonized root exudates (Fig. 2E and F).

### Effects of root exudates on rhizosphere chemical properties.

Compared with exudates from CK treatment (see Table S2 in the supplemental material), colonized root exudates had significantly decreased soil nitrate concentrations (by 6.3 to 25.2%) during the incubation period from 7 to 28 days. However, soil ammonium (NH_4_^+^) concentrations were significantly increased with P treatment and maintained concentrations that were 4.6 to 19.6% higher than those with CK treatment. Because of the greater nitrate content with CK treatment, a similar pattern was also observed in which the potential nitriﬁcation rate (PNR) in CK-treated soil was 28.1 to 47.8% higher than that of P-treated soil after 14 to 28 days of incubation. Both the CK and P treatments significantly increased soil total organic carbon (TOC) contents, and the TOC in P-treated soil was 4 to 8.1% higher than that of the CK-treated soil. Similarly, dissolved organic C (DOC) and dissolved organic N (DON) were also increased by P treatment, with levels 10.3 to 23.7% (DOC) and 9.5 to 41.2% (DON) higher in the P-treated soil than in CK-treated soil at days 14 to 28 and days 7 to 28 of incubation, respectively. In addition, the addition of colonized root exudates also significantly increased the available P (AP) (by 7.7 to 24.5%) from 7 to 28 days of incubation compared to the level with CK treatment. Furthermore, soil total N (TN), total P (TP), total K (TK), C/N, C/P, N/P, and available K (AK) levels did not differ significantly between CK and P treatments.

10.1128/mBio.00728-19.6 TABLE S2 Effects of root exudates on rhizosphere soil characteristics and nutrient concentrations. Download Table S2, DOCX file, 0.01 MB. Copyright © 2019 Xie et al. 2019 Xie et al. This content is distributed under the terms of the Creative Commons Attribution 4.0 International license.

### Effects of root exudates on *amoA* and *nifH* gene abundances.

As shown in Table 1, the copy numbers of the AOB (*amoA*) gene (ranging from 2.4 × 10^5^ to 4.9 × 10^5^ copies g^−1 ^dry soil) were significantly lower than those of the AOA (*amoA*) gene (ranging from 2.5 × 10^8^ to 7.6 × 10^8^ copies g^−1 ^dry soil) across the entire sampling time, implying that AOA may be the more important group of ammonia oxidizers in experimental soil. For AOB, compared to levels with the H_2_O treatment (ranging from 3.3 × 10^5^ to 3.9 × 10^5^ copies g^−1 ^dry soil), noncolonized root exudates (CK treatment; ranging from 3.6 × 10^5^ to 4.9 × 10^5^ copies g^−1 ^dry soil) significantly increased the AOB population by 10.6 to 30.1%; however, the colonized root exudates (P treatment; ranging from 2.4 × 10^5^ to 2.6 × 10^5^ copies g^−1 ^dry soil) significantly decreased AOB abundance by 25.2 to 36.9% during the incubation period from days 3 to 28. Further analysis found that the colonized root exudates significantly decreased AOB abundance by 47.6 to 91.4% compared to the level with CK treatment at days 3 to 28 of sampling. Similar to the AOB level, the AOA abundance was also significantly higher with the CK treatment (ranging from 3.5 × 10^8^ to 7.6 × 10^8^ copies g^−1 ^dry soil) than with any of the other treatments (both H_2_O and P treatments), and the population size was the lowest with the P treatment (ranging from 2.8 × 10^8^ to 3.9 × 10^8^ copies g^−1 ^dry soil); the AOA abundance with the CK treatment was 23.1 to 107.2% higher than that with the P treatment during the incubation period from days 3 to 28. Interestingly, the highest *nifH* gene abundance was observed with the P treatment (ranging from 6.7 × 10^5^ to 15 × 10^5^ copies g^−1 ^dry soil) though the *nifH* gene population was also increased in the noncolonized root exudates (ranging from 6.5 × 10^5^ to 8.9 × 10^5^ copies g^−1 ^dry soil) compared to the level with the H_2_O treatment (ranging from 6.4 × 10^5^ to 7.5 × 10^5^ copies g^−1 ^dry soil). The *nifH* gene abundance in P-treated soil was 35.7 to 92.5% higher than that in CK-treated soil during the incubation period from days 7 to 28.

**TABLE 1:** Effects of root exudates on the abundance of AOB, AOA, and diazotrophs in rhizosphere soil

Time point and treatment^*a*^	Abundance^*b*^		
	AOB (*amoA* copies/g dry soil) (10^5^)	AOA (*amoA* copies/g dry soil) (10^8^)	Diazotroph (*nifH* copies/g dry soil) (10^5^)
Day 0	3.25 ± 0.10	2.52 ± 0.11	6.24 ± 0.13
Day 3			
H_2_O	3.29 ± 0.14 abA	2.73 ± 0.11 aA	6.41 ± 0.11 aA
CK	3.64 ± 0.10 bA	3.46 ± 0.12 bA	6.54 ± 0.08 aA
P	2.46 ± 0.10 aA	2.81 ± 0.16 abA	6.70 ± 0.18 aA
Day 7			
H_2_O	3.25 ± 0.08 bA	3.23 ± 0.14 aB	6.48 ± 0.17 aA
CK	3.98 ± 0.07 cA	4.19 ± 0.13 bA	7.22 ± 0.30 aA
P	2.39 ± 0.08 aA	3.20 ± 0.16 aA	11.41 ± 0.21 bB
Day 14			
H_2_O	3.41 ± 0.07 bA	3.80 ± 0.17 aCD	6.22 ± 0.19 aA
CK	4.25 ± 0.12 cAB	4.86 ± 0.18 bB	7.81 ± 0.26 bA
P	2.44 ± 0.10 aA	3.88 ± 0.09 aA	15.03 ± 0.62 cD
Day 21			
H_2_O	3.76 ± 0.19 bAB	4.00 ± 0.13 bDE	6.82 ± 0.25 aAB
CK	4.89 ± 0.15 cC	5.80 ± 0.20 cC	8.25 ± 0.23 bB
P	2.56 ± 0.13 aA	3.37 ± 0.11 aBA	12.90 ± 0.28 cCD
Day 28			
H_2_O	3.93 ± 0.12 cB	4.26 ± 0.13 bEF	7.52 ± 0.20 aB
CK	3.73 ± 0.13 abA	7.62 ± 0.28 cC	8.87 ± 0.30 aB
P	2.48 ± 0.10 aA	3.68 ± 0.15 aA	12.04 ± 0.71 bBC

a H_2_O, sterile distilled water treatment; CK, noncolonized root exudate treatment; P, P. liquidambaris-colonized root exudates treatment.

b The values are means ± standard deviations from three biological replicates, with each biological replicate representing a pooled sample from at least five individual rhizosphere soil. For a column, different lowercase letters indicate significant differences among different treatments at the same sampling time, and different uppercase letters indicate significant differences among different sampling times with the same treatment. The same letters or no letters indicate no significant difference.

### Effects of root exudates on AOA and diazotroph communities.

**FIG 3:**
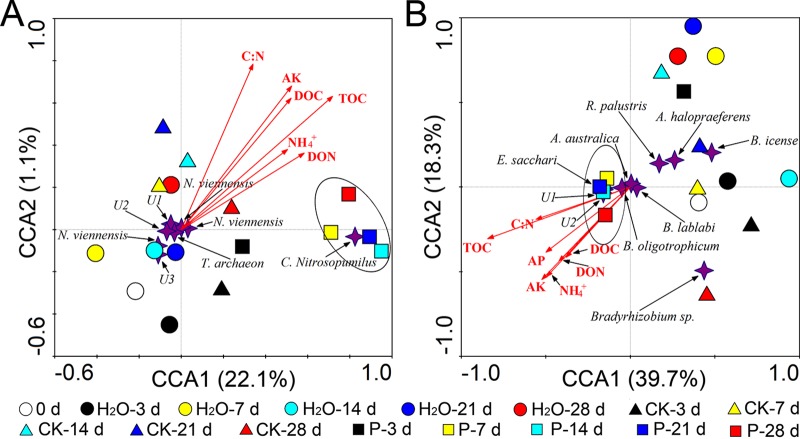
Canonical correspondence analysis (CCA) of rhizosphere soil AOA (A) and diazotroph communities (B) generated by the AOA and diazotroph DGGE patterns, respectively is shown. H_2_O, sterile distilled water treatment; CK, noncolonized root exudate treatment; P, P. liquidambaris-colonized root exudate treatment. Four-pointed star represents the identified microbial species corresponding to the excised bands from the DGGE profiles. The DGGE experiment was performed with three independent biological replicates (see Fig. S2A to D in the supplemental material), and each biological replicate representing a pooled sample from five individual rhizosphere soil. d, day.

In this study, PCR failed to amplify the *amoA* gene of AOB, which limited the analysis of the effects of root exudates on the rhizosphere AOB community structure. Cluster analysis of denaturing gradient gel electrophoresis (DGGE) band patterns of AOA *amoA* genes indicated that P. liquidambaris-colonized root exudate treatments showed low similarity with the H_2_O and CK treatment groups (Fig. S1C). We used principal-component analysis (PCA) to further characterize the distribution patterns of the AOA community and used canonical correlation analysis (CCA) to determine the impact of environmental variables on AOA community composition (23, 41). The PCA also produced similar results in which P treatment samples separately occupied an independent region from days 3 to 28 of incubation (Fig. S1E). In addition, the CCA ordination analysis demonstrated that soil nutrients, such as C/N, AK, DOC, TOC, ammonium, and DON, had significant effects on the composition of the AOA community (Fig. 3A and Fig. S2A and B). Further analysis demonstrated that “*Candidatus* Nitrosopumilus” appeared to be an important driver of the variation in the AOA community structure in soils treated with the P. liquidambaris-colonized root exudates (Fig. 3A; Fig. S1A and Table S3A). In contrast, Nitrososphaera viennensis, Thaumarchaeota archaeon, U1, U2, and U3 played key roles in the variations in the AOA community structure in the soils treated with H_2_O and noncolonized root exudates. Additionally, the richness and diversity indices (Table S4) of the AOA community were found to be significantly decreased with the P. liquidambaris*-*colonized root exudate treatment compared to that with the H_2_O and CK treatments.

10.1128/mBio.00728-19.1 FIG S1 The DGGE banding patterns of AOA (A) and diazotroph (B) communities in rhizosphere soil samples treated with peanut root exudates. The numbers indicated above the pictures are the incubation days. H_2_O, sterile distilled water treatment; CK, noncolonized root exudates treatment; P, *P. liquidambaris*-colonized root exudates treatment. Bands identiﬁed by screening of the clone libraries are designated by code number as shown in Table S3A. The DGGE experiment was performed independently in three biological replicates with similar results (each biological replicate representing a pooled sample from five individual rhizosphere soil). Cluster analysis of the rhizosphere soil AOA (C) and diazotroph (D) communities based on the produced DGGE patterns. PCA ordination diagram of rhizosphere soil AOA (E) and diazotroph (F) communities generated by the DGGE patterns. Download FIG S1, TIF file, 0.4 MB. Copyright © 2019 Xie et al. 2019 Xie et al. This content is distributed under the terms of the Creative Commons Attribution 4.0 International license.

10.1128/mBio.00728-19.2 FIG S2 CCA of rhizosphere soil AOA (A and B, remaining two replicates) and diazotroph communities (C and D, remaining two replicates) generated by the AOA and diazotroph DGGE patterns, respectively. H_2_O, sterile distilled water treatment; CK, noncolonized root exudate treatment; P, *P. liquidambaris*-colonized root exudate treatment. Four-pointed star represents the identified microbial species corresponding to the excised bands from the DGGE profiles. Download FIG S2, TIF file, 0.9 MB. Copyright © 2019 Xie et al. 2019 Xie et al. This content is distributed under the terms of the Creative Commons Attribution 4.0 International license.

10.1128/mBio.00728-19.7 TABLE S3 (A) The DNA sequence identities of selected AOA and diazotroph DGGE bands in rhizosphere simulation experiments. (B) The DNA sequence identities of selected AOA and diazotroph DGGE bands in synthetic root exudate experiments. Download Table S3, DOCX file, 0.01 MB. Copyright © 2019 Xie et al. 2019 Xie et al. This content is distributed under the terms of the Creative Commons Attribution 4.0 International license.

10.1128/mBio.00728-19.8 TABLE S4 Effects of root exudates on the richness and Shannon-Weaver indices of rhizosphere soil AOA and diazotroph communities by DGGE analysis. Download Table S4, DOCX file, 0.01 MB. Copyright © 2019 Xie et al. 2019 Xie et al. This content is distributed under the terms of the Creative Commons Attribution 4.0 International license.

On the other hand, the results of the cluster analysis and PCA ordination indicated that there were no distinct differences in the diazotroph community similarities between the H_2_O and CK treatments since these two are close together during the incubation period from days 3 to 28 (Fig. S1D and F). However, there were clear variations in the structure of the diazotroph community in soil with P. liquidambaris-colonized root exudates added compared to that with the CK treatment (Fig. S1F). The results shown in Fig. 3B and in Fig. S2C and D demonstrated that the changes in soil nutrients were positively correlated with the variations in the diazotroph community in the P-treated soil. In addition, CCA further demonstrated that some nitrogen-fixing microorganisms, such as Enterobacter sacchari, Azospirillum halopraeferens, Rhodopseudomonas palustris, and Azohydromonas australica, were responsible for driving the variations in the diazotroph community composition in the CK treatment (Fig. 3B; Fig. S1B and Table S3A). However, the increased *Bradyrhizobium* sp. populations, such as *Bradyrhizobium*, Bradyrhizobium lablabi, Bradyrhizobium oligotrophicum, and Bradyrhizobium icense, contributed greatly to the variations in the diazotroph community after colonized root exudates were added and were responsible for increasing the peanut-*Bradyrhizobium* interaction. Data in Table S4 indicate that the richness and diversity of the diazotroph community were significantly higher in P-treated soil than in CK-treated soil.

### Effects of P. liquidambaris colonization on root exudate composition.

The data shown in Table 2 indicate that *P. liquidambaris* colonization significantly increased the concentrations of TC, TN, soluble sugar, and amino acids in root exudates compared to levels in noncolonized plants, with increases of 36.8 to 49.9%, 10.3 to 43.5%, 24.2 to 48.4%, and 9.9 to 26.5%, respectively. The total organic acid concentration in the P-treated plants was 64.9 to 86.7% higher than that in the CK-treated plants, and high-performance liquid chromatograph (HPLC) analysis found that oxalic acid was considered to be the organic acid most responsive to colonization (Fig. S3). In addition, the concentrations of phenolics and flavonoids in colonized root exudates were 59.6 to 100% and 60.6 to 102.1% higher, respectively, than those in those of the CK treatment. HPLC-mass spectrometry (MS) analysis further demonstrated that the increased phenolics and flavonoids in the root exudates were mainly classified as 4-hydroxybenzoic acid, benzoic acid, coumaric acid, cinnamic acid, daidzein, genistein, and biochanin A (Fig. S4).

**TABLE 2:** Determination of TC, TN, soluble sugar, amino acids, organic acids, phenolics, and flavonoids in root exudates

Time point and treatment	Abundance (ug/plant)^*a*^						
	TC	TN	Soluble sugar	Amino acids	Organic acids	Phenolics	Flavonoids
Day 3							
CK	14.32 ± 1.65 aA	4.66 ± 0.06 aA	26.56 ± 1.62 aA	2.51 ± 0.02 aA	42.77 ± 4.56 aA	0.22 ± 0.04 aA	0.004 ± 0.001 aA
P	15.56 ± 1.16 aA	4.71 ± 0.15 aA	25.67 ± 3.51 aA	2.53 ± 0.05 aA	72.55 ± 5.80 bA	0.46 ± 0.06 bA	0.008 ± 0.001 aA
Day 7							
CK	42.97 ± 2.02 aB	8.85 ± 0.08 aB	45.39 ± 3.02 aB	3.69 ± 0.06 aB	62.55 ± 3.28 aAB	0.35 ± 0.03 aA	0.011 ± 0.001 aA
P	63.40 ± 3.00 bB	9.76 ± 0.18 bB	67.33 ± 2.57 bB	4.05 ± 0.07 bB	103.15 ± 7.60 bA	0.69 ± 0.06 bA	0.018 ± 0.002 bA
Day 14							
CK	72.34 ± 4.00 aC	14.18 ± 0.88 aB_C_	88.42 ± 7.95 aBC	6.14 ± 0.09 aC	93.13 ± 5.05 aB	0.49 ± 0.03 aA	0.016 ± 0.001 aA
P	99.00 ± 1.40 bC	18.58 ± 1.00 bC	126.10 ± 8.07 bC	7.43 ± 0.10 bC	159.54 ± 8.55 bB	0.78 ± 0.05 bA	0.032 ± 0.004 bA
Day 21							
CK	94.37 ± 5.42 aCD	17.20 ± 1.09 aBC	118.32 ± 6.11 aC	6.89 ± 0.14 aC	110.10 ± 5.40 aC	0.54 ± 0.03 aA	0.021 ± 0.002 aA
P	141.50 ± 7.51 bCD	24.68 ± 0.86 bCD	151.49 ± 5.42 bC	8.71 ± 0.13 bD	205.57 ± 9.42 bC	0.90 ± 0.04 bA	0.042 ± 0.003 bA
Day 28							
CK	122.07 ± 9.50 aDE	21.16 ± 1.01 aC	137.17 ± 7.52 aC	8.80 ± 0.14 aC	129.74 ± 12.21 aCD	0.57 ± 0.04 aA	0.023 ± 0.003 aA
P	175.84 ± 11.30 bE	28.58 ± 1.77 bCD	170.38 ± 14.52 bC	10.97 ± 0.11 bE	235.79 ± 10.38 bCD	0.99 ± 0.05 bA	0.044 ± 0.002 bA

a The values are the means ± standard deviations from three biological replicates, with each biological replicate representing a pooled sample from at least six individual plant root exudates. For a column, different lowercase letters indicate significant differences among different treatments at the same sampling time, and different capital letters indicate significant differences among different sampling time at the same treatment. The same letters or no letters indicate no significant difference.

10.1128/mBio.00728-19.3 FIG S3 HPLC chromatograms and absorbance spectra of organic acids in 7-day root exudates. (A) noncolonized root exudates. (B) *P. liquidambaris*-colonized root exudates. The experiment was repeated at least twice with similar results. Download FIG S3, TIF file, 0.2 MB. Copyright © 2019 Xie et al. 2019 Xie et al. This content is distributed under the terms of the Creative Commons Attribution 4.0 International license.

10.1128/mBio.00728-19.4 FIG S4 HPLC and HPLC-MS elution proﬁles of phenolic and flavonoid compounds in *P. liquidambaris*-colonized peanut root exudates (7 days). (A) HPLC chromatograms and absorbance spectra of phenolic and ﬂavonoid compounds in root exudates. (B) HPLC-MS spectra of 4-hydroxybenzoic acid. (C) HPLC-MS spectra of coumaric acid. (D) HPLC-MS spectra of benzoic acid. (E) HPLC-MS spectra of cinnamic acid. (F) HPLC-MS spectra of daidzein. (G) HPLC-MS spectra of genistein. (H) HPLC-MS spectra of biochanin A. The experiment was repeated at least twice with similar results. Download FIG S4, DOCX file, 0.6 MB. Copyright © 2019 Xie et al. 2019 Xie et al. This content is distributed under the terms of the Creative Commons Attribution 4.0 International license.

**FIG 4:**
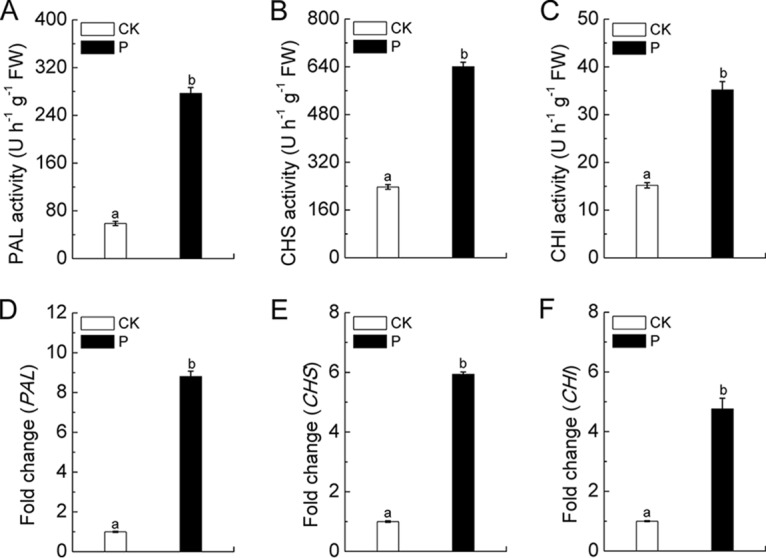
Effects of P. liquidambaris colonization on phenolic and flavonoid synthesis-related enzyme activities (A to C) and their gene expression (D to F) in peanut roots are shown. Error bars represent means ± standard deviations of three independent biological replicates, with each biological replicate representing a pooled sample from at least six individual plants. The data in columns marked by different lowercase letters are significantly different between the different treatments. FW, fresh weight.

We next investigated whether P. liquidambaris colonization could effectively increase the activities of some key enzymes involved in the biosynthesis of phenolic and flavonoid compounds (signaling compounds for symbiotic rhizobia) in peanut roots. The results shown in Fig. 4 demonstrate that P. liquidambaris symbiosis significantly increased the enzyme activities of phenylalanine ammonia-lyase (PAL), chalcone synthase (CHS), and chalcone isomerase (CHI) in roots compared to levels in the noncolonized plants. In addition, in comparison with the CK-treated roots, the roots of peanuts colonized with P. liquidambaris exhibited approximately 9-fold, 6-fold, and 5-fold increases in the expression levels of the *PAL*, *CHS*, and *CHI* genes, respectively, which ultimately contributed to the increases in biosynthesis and secretion of phenolic and flavonoid compounds in the rhizosphere soil (Fig. 4).

### Effects of root exudate components on nodulation-related biological processes in a peanut-nodulating strain.

**FIG 5:**
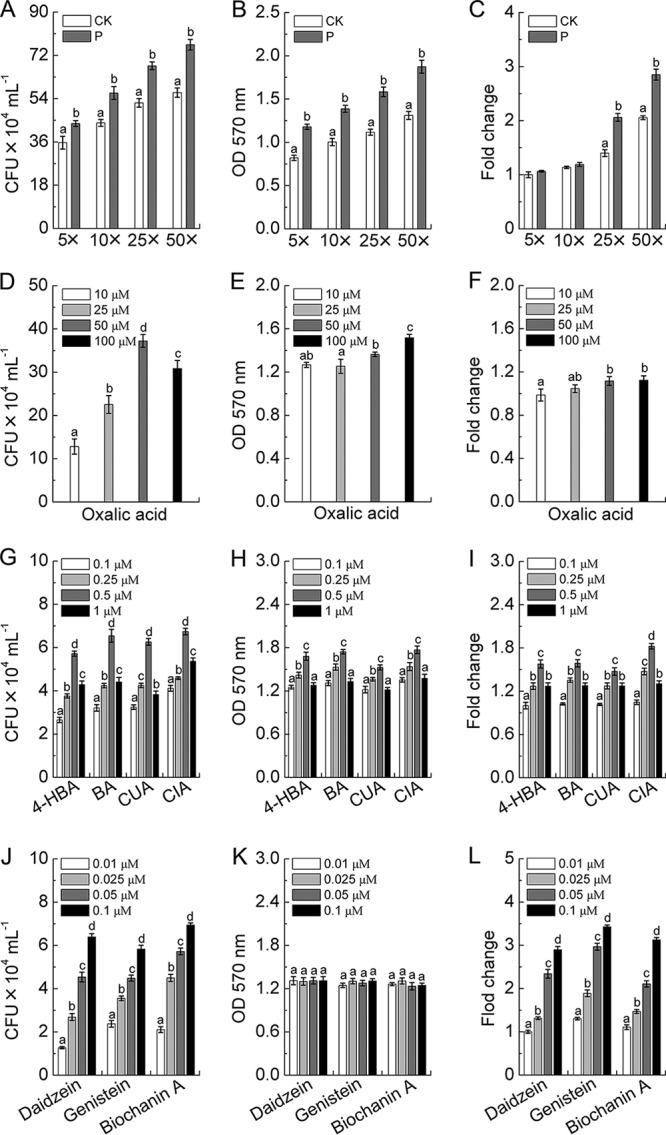
Effects of peanut root exudates (A to C), oxalic acid (D to F), phenolics (G to I), and flavonoids (J to L) on *B. yuanmingense* chemotaxis, biofilm formation, and *nodC* gene expression were determined, as indicated. Error bars represent means ± standard deviations (*n* = six biological replicates). The data in columns marked by different lowercase letters are significantly different between the different treatments, and the same letters or no letters indicate no significant difference. CK, noncolonized root exudate; P, P. liquidambaris-colonized root exudate; 4-HBA, 4-hydroxybenzoic acid; BA, benzoic acid, CUA, coumaric acid; CIA, cinnamic acid.

The results shown in Fig. 5A to C indicate that, compared with CK treatment levels, a gradient of concentrations of root exudates colonized with P. liquidambaris significantly increased the chemotaxis and biofilm formation of Bradyrhizobium yuanmingense. They also effectively enhanced the expression of the *nodC* gene at the 25-fold and 50-fold concentration gradients. Further studies demonstrated that as the concentration of the oxalic acid increases, the chemotaxis of B. yuanmingense was first increased and then decreased; however, biofilm formation and *nodC* gene transcription continually increased slightly (Fig. 5D to F). In addition, the application of phenolic acids (4-hydroxybenzoic, benzoic, coumaric, and cinnamic acids) gradually increased the chemotaxis, biofilm formation, and *nodC* gene expression of B. yuanmingense, which exhibited the highest level at a concentration of 0.5 μM but was slightly inhibited at 1 μM (Fig. 5G to I). However, results shown in Fig. 5J to L demonstrate that the flavonoid compounds (daidzein, genistein, and biochanin A) could significantly enhance the chemotaxis and *nodC* gene transcription of B. yuanmingense within the detected concentration range.

### Effects of root exudates on rhizosphere microbial metabolic pattern.

**FIG 6:**
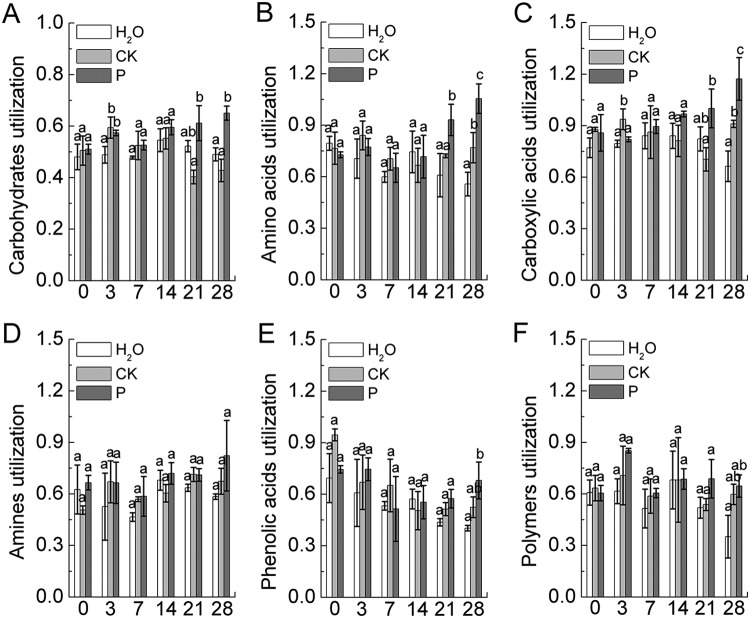
(A to F) Relative utilization ratios of six groups of carbon sources by the rhizosphere soil microbes on Biolog EcoPlates (plate incubation for 72 h) at sampling of 0-, 3-, 7-, 14-, 21-, and 28-day soils, as indicated. Data are means ± standard deviations of three biological replicates, with each biological replicate representing a pooled sample from at least five individual rhizosphere soils. Different lowercase letters indicate significant differences among treatments, and the same letters or no letters indicate no significant difference.

The rhizosphere soil metabolic patterns were detected to assess the metabolic mode of rhizosphere microbes for carbon source utilization. As shown in Fig. 6, the addition of root exudates from different sources differentially affected the carbon source utilization patterns in the rhizosphere soil. P. liquidambaris-colonized root exudates significantly increased the utilization levels of carbohydrates, amino acids, and carboxylic acids by 51.8%, 29.1%, and 42.2% at 21 days and by 52.0%, 37.1%, and 28.7% at 28 days of incubation, respectively, compared with the CK treatment levels (Fig. 6A to C). However, the results shown in Fig. 6E and F indicate that no significant differences were observed in the carbon source utilization rates of phenolic acids or polymer compounds among the H_2_O, CK, and P treatments during the gradual addition of root exudates from days 0 to 28 of incubation.

### Effects of SRE on nodulation and N_2_ fixation.

**FIG 7:**
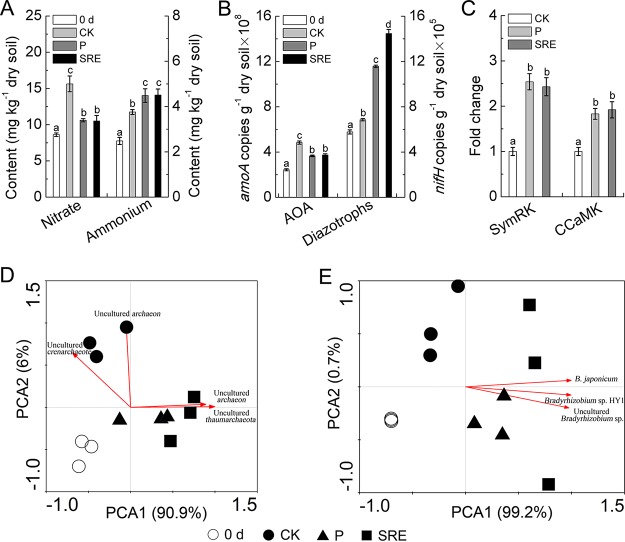
(A) Effects of synthetic root exudates (SRE) on rhizosphere soil nitrate (NO_3_^−^) and ammonium (NH_4_^+^) concentrations. (B) Effects of SRE on rhizosphere soil AOA and diazotroph abundance. (C) Effects of SRE on symbiosis-related gene expression (*SymRK* and *CCaMK*). Principal-component analysis (PCA) of the effects of SRE on AOA (D) and diazotroph (E) community structures are shown. All experiments were performed independently in three biological replicates, with each biological replicate representing a pooled sample from at least six individual plants or six individual plant rhizosphere soils. The data in columns marked by different lowercase letters are significantly different between the different treatments, and the same letters or no letters indicate no significant difference.

Field pot experiments demonstrated that synthetic root exudate (SRE) addition significantly decreased the concentration of rhizosphere soil nitrate but increased ammonium content, which was similar to the results of the P treatment (Fig. 7A). Similarly, we further observed that SRE and P treatments effectively decreased the abundance of the AOA *amoA* gene in the rhizosphere, while the abundance of the diazotroph *nifH* gene was significantly increased compared to that with the CK treatment (Fig. 7B). PCA found that the AOA community structures in the P and SRE treatments were similar and cooccupied a separate region (Fig. 7D) (analysis of similarities [ANOSIM] *R *=* *0.849; *P = *0.001). Further analysis indicated that uncultured *Thaumarchaeota* and *Archaeon* (Table S3B) may play an important role in the variations in the AOA community after P. liquidambaris colonization and SRE addition. On the other hand, the addition of SRE significantly changed the diazotroph community, which was also found to be similar to the effects produced by P. liquidambaris colonization because the P and SRE treatments cooccupied the right region of the PCA ordination plot (Fig. 7E) (ANOSIM *R *=* *0.920; *P = *0.001). The correlation analysis found that *Bradyrhizobium* sp. populations, such as Bradyrhizobium japonicum, *Bradyrhizobium* sp. strain HY1, and uncultured *Bradyrhizobium* sp. (Table S3B), also contributed greatly to affect the diazotroph community in P. liquidambaris colonization and SRE treatments.

As shown in Fig. 7C, the addition of SRE significantly increased the transcript levels of the peanut-*Bradyrhizobium* symbiosis-related genes *SymRK* and *CCaMK*, which was consistent with the results for peanut roots colonized with P. liquidambaris, indicating that the change in the peanut root exudates caused by P. liquidambaris colonization was responsible for enhancing the peanut-*Bradyrhizobium* interaction. Further analysis demonstrated that nodulation and N_2_ fixation were also significantly increased in the P. liquidambaris colonization and SRE treatments (Table 3). In addition, we observed that the P and SRE treatments effectively increased peanut growth parameters, such as shoot and root dry weight.

**TABLE 3:** Effects of P. liquidambaris colonization and synthetic root exudate addition on peanut nodulation and N_2_ fixation^*a*^

Treatment	No. of nodules/plant	Total nodule wt (mg/plant)	Shoot N content (mg/plant)	N_2_ fixation (mg/plant)	Shoot dry wt (mg/plant)	Root dry wt (mg/plant)
CK	30.00 ± 3.46 a	59.49 ± 4.77 a	15.33 ± 0.88 a	5.78 ± 0.17 a	0.81 ± 0.04 a	0.34 ± 0.02 a
P	46.33 ± 1.53 b	88.75 ± 6.01 b	28.55 ± 1.80 b	10.09 ± 0.82 b	1.00 ± 0.06 b	0.39 ± 0.03 a
SRE	43.33 ± 1.53 b	83.87 ± 4.51 b	34.25 ± 1.63 c	12.05 ± 0.81 b	0.99 ± 0.04 b	0.40 ± 0.03 a

a The values are means ± standard deviations from three biological replicates, with each biological replicate representing a pooled sample from at least six individual plants. For a column, different lowercase letters indicate significant differences among different treatments, and the same letters or no letters indicate no significant difference.

## DISCUSSION

A consecutive 2-year field plot experiment showed that low levels of N fertilizer significantly increased peanut nodulation and N_2_ fixation, which was consistent with previous findings that high-N applications in leguminous monoculture systems inhibited nodulation and N_2_ fixation (11). However, root colonization with the endophytic fungus P. liquidambaris can significantly increase nodulation and N_2_ fixation capacities of peanuts, even under high-N conditions, implying that a facilitative effect of P. liquidambaris symbiosis on peanut nodulation and N_2_ fixation is present regardless of soil N levels. In addition, we observed that low-N soil effectively increased the amount of P. liquidambaris colonization in roots, and this double-superposition effect might be further beneficial to improving nodulation and N_2_ fixation, which will ultimately enhance the N uptake of peanuts under low-N soil conditions.

The two-layer potted experiment demonstrated that the P. liquidambaris*-*colonized root exudates were an essential factor in this facilitative effect and provided direct evidence that the specific root exudate secretion caused by P. liquidambaris symbiosis might be important for improving nodulation and N_2_ fixation. Many studies have demonstrated the use of beneficial exogenous microorganisms to target host root exudates as a promising method to increase crop productivity (24, 39, 40, 42). However, efficient colonization of the exogenous microbial agents is an indispensable condition that must be met in order for them to exert their potential ecological functions. P. liquidambaris can steadily colonize peanut roots in the field, and this confers the ability of P. liquidambaris to continuously stimulate the secretion of specific root exudates by peanuts. Indeed, P. liquidambaris colonization significantly changed the secretion of organic compounds in the root exudates and increased the concentrations of soluble sugars, amino acids, organic acids, phenolics, and flavonoids. At present, increasing evidence suggests that root exudates can regulate host PRFE, including nutrient availability, microbial community, and plant-microbe interactions (13, 30, 31, 43). Therefore, whether the secretion of specific root exudates induced by P. liquidambaris symbiosis can improve PRFE and whether this effect contributes to the increase in nodulation and N_2_ fixation are questions worthy of investigation in this study.

Nutrient availability in the rhizosphere soil is one of the most important factors affecting the communication between plants and soil (44). For legumes, the concentrations of ammonium (NH_4_^+^) and nitrate (NO_3_^−^) in the rhizosphere played a very important role in regulating nodule formation and development (10, 32, 33). Previous studies have demonstrated that the development and N_2_ fixation activity of root nodules are known to be significantly suppressed when nodulated roots are exposed to a high concentration of nitrate (10). This study found that the addition of P. liquidambaris-colonized root exudates significantly reduced the concentration of nitrate in the rhizosphere soil, and this approach might be conducive to improving nodulation initiation and development. However, in contrast, ammonium in the rhizosphere soil significantly increased after application of colonized root exudates. These changes may be mainly attributed to the changes in abundance and community composition of ammonia-oxidizing archaea (AOA) in the colonized-root exudate treatment because AOA played a key role in driving the conversion of ammonium to nitrate in the present study. In addition, a previous study demonstrated that ammonium might be a more efficient N source in the rhizosphere as it resulted in higher nodule dry weight, total N accumulation, and total N_2_ fixation (45), and as a result, ammonium should be considered another important factor in increasing peanut nodulation and N_2_ fixation.

It is well known that legume nodulation initiation and development comprise a process that heavily consumes energy; therefore, the growth state and carbohydrate availability of host plants might be responsible for the normal development of nodules and the ability to fix N_2_ (24). Soil nutrients are the most critical limiting elements for plant growth and carbohydrate storage (44, 46). Though P. liquidambaris-colonized root exudates significantly decreased the concentration of soil nitrate (this is beneficial for increasing legumes nodulation by enhancing specific nodule signaling pathways) (10), they effectively increased the concentrations of many other available nutrients in the rhizosphere soil, such as ammonium, DOC, DON, AP, and AK, which may contribute to promote plant growth and be helpful for further stimulating nodulation and N_2_ fixation to some extent. This was further confirmed by the synthetic root exudate experiment in which root exudates effectively increased the dry weight of peanut shoots and roots. A previous study also demonstrated that P. liquidambaris colonization could increase leaf photosynthetic activity and the soluble sugar accumulation of peanuts (25). However, the detailed mechanisms involved in the enhancement of the carbohydrate of host accumulation caused by endophytic fungus P. liquidambaris colonization need further investigation.

Nodule formation and N_2_ fixation in legumes begin with the interaction of legumes and rhizobium, so the abundance and diversity of nodulation-specific rhizobia in the rhizosphere soil should be considered the most fundamental element in determining the initiation of nodules (12, 26). Traditionally, it has been thought that peanuts form effective nodules with slow-growing rhizobia belonging to the genus *Bradyrhizobium* (12, 16). A previous study revealed hyperdiverse peanut *Bradyrhizobium* bacteria in different geographical locations using nitrogenase (*nifH*) gene probes (47). Here, the quantitative PCR (qPCR) and DGGE analyses showed that the addition of P. liquidambaris-colonized root exudates significantly increased the abundance, diversity, and community composition of the *nifH* gene in the rhizosphere soil. Surprisingly, the increased diazotroph (*nifH*) community mostly belonged to the *Bradyrhizobium* genus, including *Bradyrhizobium* sp., *B. lablabi*, *B. oligotrophicum*, and *B. icense*, which provided direct evidence for enhancing peanut nodulation and N_2_ fixation in the aspect of rhizobial symbioses.

Previous studies have shown that the *Rhizobium* community in the rhizosphere soil undergoes changes in response to phenolic and flavonoid compounds when they accumulate in the soil, which provides a competitive advantage for nodulation by selecting for rhizobial strains (38, 48). After symbiosis with P. liquidambaris in peanut roots, the activities of key enzymes (PAL, CHS, and CHI) in phenolic and flavonoid biosynthesis were significantly increased, which was confirmed by measuring the transcript levels of their corresponding genes. Therefore, the increased phenolics and flavonoids in P. liquidambaris-colonized root exudates should be considered an important reason to select for the rhizobium-dominated microbiome in the peanut rhizosphere. In addition, host root-excreted phenolic and flavonoid compounds acted as important signaling molecules during the expression of various symbiotic plasmid-encoded nodulation genes, as well as a range of soluble phenolic and flavonoid compounds involved in rhizobial recognition and nodule morphogenesis in *Arachis hypogaea* L. plants (38, 49). Our results also demonstrated that P. liquidambaris-colonized root exudates and their derived components (organic acid, phenolics, and flavonoids compounds) significantly increased the chemotaxis, biofilm formation, and *nodC* gene expression of the peanut-nodulating *Bradyrhizobium* strain. Metabolic pattern analysis showed that the phenolics and polymers are difficult to use as carbon sources in the rhizosphere soil after addition of P. liquidambaris-colonized root exudates. This indicated that phenolics and flavonoids are more likely to accumulate in the rhizosphere soil when peanuts are colonized by P. liquidambaris, which may further improve the peanut-*Bradyrhizobium* symbiosis interaction. In addition, the increase of the enzyme activities of PAL, CHS, and CHI might contribute to enhance the defense resistance of the host to pathogens (50), which would further explain why P. liquidambaris colonization decreased the incidence of peanut disease in our previous field study (9). This may help to promote peanut growth and thus be beneficial for increased peanut nodulation and N_2_ fixation.

**FIG 8:**
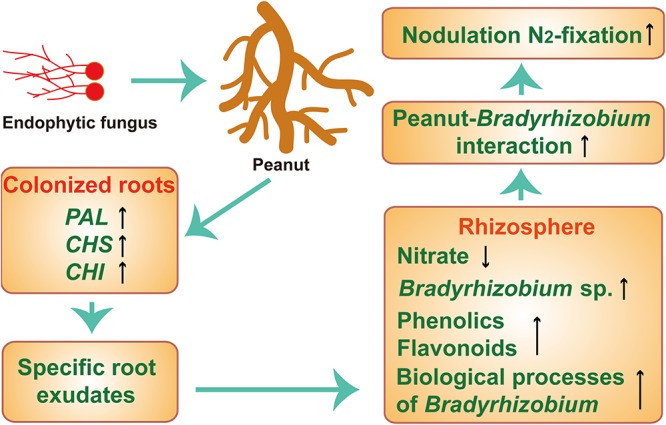
The secretion of specific root exudates caused by endophytic fungus P. liquidambaris colonization drives increases in peanut nodulation and N_2_ fixation. P. liquidambaris colonization induces significant upregulation of PAL, CHS, and CHI gene expression in roots, which, at least partially, led to the production of specific peanut root exudates. The secretion of specific root exudates from P. liquidambaris-colonized roots can effectively decrease nitrate (NO_3_^−^) concentration in the rhizosphere soil, increase rhizosphere soil *Bradyrhizobium* sp. quantity and diversity, increase rhizosphere soil phenolic and flavonoid accumulation, and increase the nodulation-related biological activities of rhizosphere *Bradyrhizobium* strains, which significantly enhance peanut-*Bradyrhizobium* interactions. Up arrow, significant upregulation; down arrow, significant downregulation.

To prove the findings mentioned above, a synthetic root exudate experiment was performed under the field pot conditions. The addition of synthetic root exudates significantly increased peanut nodulation and N_2_ fixation, and the promoted effects were similar to those in plants only colonized with P. liquidambaris, indicating that the derived root exudates from P. liquidambaris colonization, indeed, have a crucial role in increasing peanut nodulation and N_2_ fixation. Similar to results of the rhizosphere simulation experiments, both P. liquidambaris colonization and synthetic root exudates significantly decreased the concentration of rhizosphere nitrate but increased the *nifH* community abundance and diversity. This change in the *Bradyrhizobium* population has also been proven to be responsible for driving the change in the community structure of diazotrophs in the rhizosphere. In addition, the transcript levels of the symbiosis-related genes *SymRK* and *CCaMK* increased significantly in response to synthetic root exudates, which was also similar to results in P. liquidambaris -colonized plants. Therefore, this study demonstrates that the increases in nodulation and N_2_ fixation are attributed to the impact of specific root exudates of P. liquidambaris colonization on PRFE (Fig. 8). However, in this study, the root exudate treatments may not be fully reflective of the effects of root exudates since in some of these experiments roots were actually absent. Therefore, in a subsequent study, we will further explore the direct impact of peanuts colonized by P. liquidambaris on the rhizosphere microenvironment under the pot experiment or actual field conditions and also investigate whether this improvement is correlated to the increase of nodulation and N_2_ fixation.

### Conclusion.

Endophytic fungi play an important role in balancing the ecosystem and boosting host growth; however, the underlying mechanisms remain poorly understood. Here, we found that endophytic fungal colonization with P. liquidambaris significantly increased the productivity, nodulation, and N_2_ fixation of peanuts through the secretion of specific root exudates. We provide a reasonable mechanism explaining how P. liquidambaris promotes peanut nodulation and N_2_ fixation, where the specific root exudates produced by P. liquidambaris colonization decrease rhizosphere soil nitrate (NO_3_^−^) and increase the population and biological activities of peanut-nodulating-related *Bradyrhizobium* strains, which is beneficial to enhancing the peanut-*Bradyrhizobium* symbiotic interaction. Our study provides reliable empirical evidence to show the mechanism of how an exotic endophytic fungus drives an increase in nodulation and N_2_ fixation, which will be helpful in erecting a resource-efficient and sustainable agricultural system.

## MATERIALS AND METHODS

### Field plot experiment 1 (field peanut nodulation and N_2_ fixation assay).

A 2-year field plot experiment was conducted in 2015 and 2016 at the Nanjing Normal University Botanical Garden (Jiangsu province, China; 28°13′N, 116°55′E). Soil characteristics have been reported previously (9). The endophytic fungus P. liquidambaris was grown in potato dextrose broth medium (200 g liter^−1^ potato extract, 20 g liter^−1^ glucose, pH 7.0) for 48 h at 28°C on a shaker at 180 rpm. Fungal mycelia were collected by filtering and were repeatedly washed with sterile distilled water and then resuspended in sterile distilled water to serve as the fungal inoculum. Guanhua-5 peanut seeds (13) were soaked for 5 min in 70% ethanol, followed by five washes with sterile distilled water and further surface disinfection in 1% (vol/vol) sodium hypochlorite for 3 min and then by another round of washing. The surface-sterilized seeds were randomly divided into two groups and transferred to petri dishes (20-cm diameter; 20 seeds per dish). For the P. liquidambaris colonization group (P treatment), 100 ml of the P. liquidambaris inoculum containing 124.7 mg of mycelia (dry weight) was added to each dish. The noncolonized group (CK treatment) was treated with 100 ml of sterile distilled water. After a pregermination period of 3 days in the dark at 28°C, the germinated seeds were transplanted into cultivation boxes (28 cm long, 18 cm wide, and 10 cm high) containing sterile vermiculite for raising seedlings. After 5 days of cultivation, seedlings at similar development stages were transplanted into field plots (3 m by 4 m). Peanut plants were randomly arranged in a 2-by- 2 factorial design, in which the main effects were the P. liquidambaris colonization and the amount of N applied (the plot had two N application rates, 0 and 140 kg N ha^−1^, applied as urea). In this plot experiment, 10 rows of peanuts were planted with a 0.4-m interrow distance and a 0.2-m interplant distance. The experimental plots were separated by a 1- m-wide buffer zone, and watering and weeding were performed manually when necessary. The peanuts were planted on 25 April 2015 and 25 April 2016 and harvested on 15 August 2015 and 20 August 2016, respectively. No crops were planted in field plots from September to the following April.

The plants were collected after 10, 30, and 60 days of planting. Because the colonization process of P. liquidambaris in peanut roots has been shown to be dynamic (9, 16), only the amount of P. liquidambaris colonization was measured to verify successful colonization in this study. A fragment of the P. liquidambaris-specific internally transcribe spacer (ITS) locus was amplified with previously designed primers Bf1 and Br1 (see Table S1 in the supplemental material) to confirm endophytic fungal colonization level. The quantitative real-time PCR (qPCR) mixture and thermal cycling conditions were consistent with our previous study (20), and the colonization concentration was expressed as the number of P. liquidambaris-specific ITS copies per nanogram of total genomic DNA. The active nodules were excised from the roots (60 days) with scalpels to measure the nodule number and total nodule weight. The aboveground shoot samples were ground to a fine powder, and the N concentrations were determined using the Kjeldahl method (51). The derived N_2_ fixation from P. liquidambaris colonization was estimated by the N difference method (16), in which the presumptive amount of N_2_ fixation was calculated by subtracting the nitrogen level of uninoculated peanut from that of inoculated peanut plants. After harvest, the peanut pod yield was also measured.

10.1128/mBio.00728-19.5 TABLE S1 Primers used for the real-time PCR and PCR-DGGE analysis. Download Table S1, DOCX file, 0.01 MB. Copyright © 2019 Xie et al. 2019 Xie et al. This content is distributed under the terms of the Creative Commons Attribution 4.0 International license.

### Greenhouse experiment 1 (peanut nodulation as affected by root exudates).

To collect peanut root exudates, the above P. liquidambaris-colonized (P treatment) and noncolonized (CK) seedlings were transplanted into plastic pots (15 cm in diameter and 13 cm high) containing sterile vermiculite, with one seedling per pot. Hoagland’s nutrient solution was applied every 2 days, and sterile distilled water was added when needed. After 10 days of growth, 20 seedlings from each treatment were washed and then transferred into 200 ml of sterile distilled water. Plantlets were maintained in a biochemical incubator at 28°C for 2 h to collect root exudates. The collected peanut root exudates were filtered by a double layer of Whatman no. 1 filter paper and used in the following bioassays.

To investigate whether the root exudates from seedlings colonized with P. liquidambaris can affect peanut nodulation, root exudate addition bioassays were performed. Bradyrhizobium yuanmingense was obtained from Culture Collection of Beijing Agricultural University (CCBAU21353); it had been previously isolated from the surface-sterilized nodules of peanut and cultured in yeast mannitol broth medium (10 g liter^−1^ d-mannitol, 3 g liter^−1^ yeast extract, 0.1 g liter^−1^ MgSO_4_, 0.1 g liter^−1^ NaCl, 0.25 g liter^−1^ K_2_HPO_4_, 0.25 g liter^−1^ KH_2_PO_4_,) in an orbital shaker at 180 rpm and 28°C for 6 days (16). The bacterial cells were centrifuged at 4,000 × *g* for 10 min, resuspended in sterile distilled water, and then diluted to 10^8^ cells liter^−1^. The surface-disinfected peanut seeds were inoculated with a suspension of B. yuanmingense and sown into cultivation boxes containing sterile vermiculite for raising seedlings. After 5 days of cultivation, seedlings at similar developmental stages were transplanted into a two-layered pot system (52), in which the upper pot was filled with 400 ml of sterile vermiculite and the lower pot was filled with 1 liter of nutrient solution (11). After 3 days, nitrate (NO_3_^−^) was applied to the nutrient solution at 0, 1, or 2 mM. Ten milliliters of B. yuanmingense suspension was inoculated into each upper pot. In addition, each day, 10 ml of collected root exudate from peanuts was separately added to serve as the root exudate treatment (P and CK treatments, respectively), and sterile distilled water was added as the control (H_2_O treatment). Peanut plants were cultured in this two-layered pot system for 28 days. After harvesting, the peanut nodulation and N_2_ fixation parameters were evaluated, and the transcript levels of peanut-*Bradyrhizobium* symbiosis-related genes (*SymRK and CCaMK*) were determined (16).

### Greenhouse experiment 2 (plant rhizosphere simulation experiment).

**(i) Experimental methods.** Similarly, the P. liquidambaris-colonized (P treatment) and noncolonized (CK) seedlings at a consistent developmental stage in cultivation boxes were transplanted in pots containing sterile vermiculite with one seedling per pot. All pots were randomly distributed in the growth chamber with 70% relative humidity at 28°C with 16 h of light and at 25°C with 8 h of dark. Hoagland’s nutrient solution was added every 2 days, and sterile distilled water was added when needed. Each day after transplantation (from day 1 to day 28) the peanut seedlings were sampled, and the root systems were carefully washed with sterile distilled water to remove the vermiculite. To collect the root exudates, seedlings were transferred into a sterile 100-ml glass container with 50 ml of sterile distilled water and maintained in a biochemical incubator (mentioned above) for 24 h. The collected root exudates (about 50 ml) were filtered by a double layer of Whatman no. 1 filter paper and immediately freeze-dried. The lyophilized root exudate powder was dissolved in sterile distilled water to make concentrated extracts of 1 ml/seedling for the bioassay and the analysis of root exudates. In this study, a total of 28 root exudate samples were collected, corresponding to the daily samples over the 28-day growth of the seedlings. Each treatment included triplicates for each sampling time.

Experimental soils (consecutively cropped with peanuts for 10 years) were collected from an agricultural field at the Ecological Experimental Station of Red Soil at the Chinese Academy of Sciences (China; 28°13′N, 116°66′E). The selection of the collection soil was deliberate as obstacles in continuous peanut cropping are particularly widespread in the hilly red soil region of southern China. The soil was classified as Udic Ferrosol (FAO classiﬁcation, 1998). Soil was taken from the surface layer (0 to 20 cm) and sieved (2 mm). After removal of organic debris, the soil was divided into two parts for air drying to determine the physical and chemical properties of soil and for storage at 4°C for all other experiments in this study. The basic physicochemical properties of soil were as follows: organic matter, 9.4 mg kg^−1^; total N, 0.7 g kg^−1^; total P, 0.4 g kg^−1^; total K, 11.5 g kg^−1^; ammonium (NH_4_^+^), 2.4 mg kg^−1^; nitrate, 8.3 mg kg^−1^; available P, 12.4 mg kg^−1^; available K, 113.1 mg kg^−1^; pH 5.7 (soil to water ratio, 1:2.5).

Plant rhizosphere simulation experiments were performed as previously reported (13, 53). Briefly, 100 g of moist soil was placed in glass cylinders (90 mm in diameter and 20 mm high). A cellulose filter (Whatman no. 41; 90-mm diameter) was placed on top of the soil and treated immediately with 2 ml of sterile distilled water using a pipette to uniformly adhere to the surface of the soil. To simulate the practical dynamic effects of root exudate release over the 28-day incubation period, 1 ml of root exudate aliquots collected from the treated seedlings above corresponding to days 1 through 28 was added each day dropwise. Thus, the root exudate addition was performed a total of 28 times. On day 0, 1 ml of sterile distilled water was added. The control soil received an equivalent amount of sterile distilled water. Each cylinder was placed in a 1-liter airtight jar, which was then maintained in an incubator, and the culture conditions were consistent with those described above. The jars were aerated once per day for 1 h, and the soil moisture was maintained at 60% of the water-holding capacity.

Three replicate test units per treatment were sampled at 0, 3, 7, 14, 21, and 28 days of incubation. The layer at 0 to 2 mm below the surface of the ﬁlter was considered the simulated rhizosphere of the soil (54) and served as the sample source. The rhizosphere microbial metabolic pattern was analyzed immediately, and other soil samples were stored at −20°C until further measurement of the microbial communities and chemical properties.

**(ii) Rhizosphere soil chemical properties analysis.** Soil ammonium and nitrate were measured by extraction from the soil with a 0.01 mol liter^−1^ CaCl_2_ solution (1:10, wt/vol) and determination with a flow injection autoanalyzer (FLA Star 5000 Analyzer; Foss, Denmark) (23). Soil pH was determined with a glass electrode using a soil-to-water ratio of 1:2.5. Soil total organic C (TOC) and total N (TN) were determined by dichromate oxidization and Kjeldahl digestion methods, respectively (55). Soil total phosphorus (TP) and potassium (TK) were determined by digestion with HF-HClO_4_ and molybdenum-blue colorimetry and ﬂame photometry, respectively (56). Soil dissolved organic C (DOC) and dissolved organic N (DON) were determined as previously described (57). The Olsen and acetate extract-flame photometer methods were employed to detect available P (AP) and K (AK), respectively (55). A chlorate inhibition method was used to determine the potential nitriﬁcation rate (PNR) as previously reported (23).

**(iii) Soil DNA extraction and qPCR assay.** The total soil DNA was extracted from 0.5 g of the soil subsamples using a FastDNA spin kit for soil (MP Biomedicals, Santa Ana, CA, USA) according to the manufacturer’s protocols. DNA was then quantified using a UV spectrophotometer (Bio Photometer; Eppendorf, Germany). qPCR was performed with appropriate primers (Table S1) to determine the relative abundance of rhizosphere soil AOB (*amoA*), AOA (*amoA*), and diazotroph (*nifH*) in each of the sampled soils. The qPCR method was consistent with methods described in our previous study (23), and the abundances of the *amoA* (AOB), *amoA* (AOA), and *nifH* genes were expressed as the number of gene copies per gram of dry soil.

**(iv) DGGE.** PCRs were performed using an Eppendorf C1000 Touch Thermal Cycler (Eppendorf, Hamburg, Germany), and the *amoA* (AOB), *amoA* (AOA), and *nifH* genes were amplified with primer pairs containing a GC clamp in the one primer (Table S1). The 50-μl reaction mixtures consisted of 5 μl of 10× PCR buffer (Mg^2+^-free), 4 μl of 2.5 mM deoxynucleoside triphosphates (dNTPs), 0.5 μl of *Taq* polymerase (5 U μl^−1^; TaKaRa Biotechnology, Japan), 1 μl of each primer at 50 mM, and 1 μl of DNA template (10 to 15 ng). To amplify bacterial and archaeal *amoA* gene fragments, the touchdown PCR strategy used included 94°C for 3 min, followed by a touchdown procedure (94°C for 40 s, annealing for 45 s at temperatures decreasing from 60 to 55°C during the first 10 cycles, and with a final extension step at 72°C for 1 min). This was followed by 35 additional cycles of 94°C for 40 s, 55°C for 45 s, 72°C for 1 min, and, finally, an extension at 72°C for 10 min. For the *nifH* gene, the PCR strategy was initial denaturation at 95°C for 3 min, 35 cycles of 94°C for 1 min, 55°C for 1 min, and 72°C for 1 min, with a final extension at 72°C for 10 min (20, 23, 58). Denaturing gradient gel electrophoresis (DGGE) analyses were carried out as follows: the PCR amplicons of *amoA* (AOB) and *amoA* (AOA) genes were separated on a 6% (wt/vol) acrylamide-bisacrylamide gel with denaturing gradients of 40 to 70% and 20 to 55%, respectively (the 100% denaturant contained 7 M urea and 40% formamide). The PCR product of the *nifH* gene was separated on an 8% (wt/vol) acrylamide-bisacrylamide gel using a denaturing gradient of 40 to 70%. After being run for 16 h at 100 V, the DGGE gels were stained with SYBR green I (Invitrogen Molecular Probes, Eugene, OR, USA) for 30 min and scanned by a GelDOC-ItTS imaging system (Ultra Violet Products, Upland, CA, USA) following UV transillumination.

**(v) Cloning of PCR products and sequencing.** For sequencing, selected bands were excised from DGGE gels and transferred into a sterile Eppendorf tube containing 20 μl of sterile distilled water. The DNA was eluted and incubated overnight at 4°C. PCR was performed with the same primer sets used in the DGGE analysis (without the GC clamp) and purified using an agarose gel DNA puriﬁcation kit (TaKaRa Co., Dalian, China). The ampliﬁed fragments were cloned into the pEASY-T1 vector (TransGen, Beijing, China), and chemically competent Escherichia coli cells were transformed with the plasmids according to the manufacturer’s protocols (TransGen Biotech, Beijing, China). Transformants were randomly picked by blue-white selection, and the cloned DNA fragments were sequenced using an ABI 3730 automated sequencer (Invitrogen, Shanghai, China). The obtained sequences were compared with sequences in the BLAST/NCBI database (https://www.ncbi.nlm.nih.gov/BLAST).

### Greenhouse experiment 3 (root exudate analysis and functions).

**(i) Analysis of root exudate, phenolic and flavonoid synthesis-related enzyme activities, and gene expression.** The collected peanut root exudates were filtered through a 0.22-μm-pore-size filter prior to the bioassays. Total C and N were determined using ae Vario EL III Elemental analyzer (Elementar Analysensysteme GmbH, Germany). Soluble saccharides were determined by the anthrone colorimetry method (59). The separation and quantiﬁcation of organic acids and amino acids were performed using high-performance liquid chromatography (HPLC) (HP1100; Agilent, USA) according to the method described in our previous studies (23, 43). In addition, phenolics and flavonoids in root exudates were detected and identiﬁed by HPLC-MS (mass spectrometry) according to the previous methods with several modifications (43, 60). The analytical conditions were as follows: chromatographic column, XDB-C_18_ (4.6 by 250 mm; Agilent, USA); temperature, 30°C; flow rate, 0.5 ml/min; detector wavelength, 280 nm; injection volume, 20 μl. Acetic acid (2%, vol/vol) solution (A) and methanol (B) were used as mobile phases with a gradient elution (5% B at 0 min, 15% B at 10 min, 50% B at 30 min, 60% B at 40 min, 95% B at 50 min, and holding to 60 min). MS was performed using a 6460 Triple Quad LC/MS operated in the electrospray ionization (ESI) mode with a negative/positive polarity and scanned by normal mass range from 50 to 500 *m/z*. The activities and transcript levels of key phenolic and flavonoid synthesis-related enzymes in peanut roots were measured 10 days after transplantation from the cultivation boxes. The roots were carefully washed with sterile distilled water to remove vermiculite, and the enzyme activities of PAL (phenylalanine ammonia-lyase), CHS (chalcone synthase), and CHI (chalcone isomerase) were determined as previously described (24, 61). In addition, the transcript levels of the *PAL*, CHS, and *CHI* genes in peanut roots were evaluated by qPCR, as mentioned above (Table S1).

**(ii) Detection of root exudate component effects on biological processes in a peanut-nodulating strain.** To determine the effects of the root exudates and the identified organic acid, phenolic, and flavonoid compounds on nodulation-related biological processes in the peanut-nodulating *Bradyrhizobium* strain, the chemotaxis ability and biofilm formation of B. yuanmingense were determined as previously reported (62, 63). For root exudate treatment, the 50 ml of collected root exudate (mentioned in the description of greenhouse experiment 2) was first concentrated to 0.5 ml as the 100-fold-concentrated root exudate (100×), and then gradually diluted to 50×, 25×, 10×, and 5× to serve as the final test concentrations. In addition, four concentration gradients of organic acid (10, 25, 50, and 100 μM) and phenolic (0.1, 0.25, 0.5, and 1 μM) and flavonoid (0.01. 0.025. 0.05, and 0.1 μM) compounds were, respectively, used in this bioassay. Chemotaxis ability and biofilm formation of B. yuanmingense were expressed as the number of CFU (×10^4^ ml^−1^) and optical density at 570 nm (OD_570_) (absorption value), respectively. In addition, the expression level of the *nodC* gene (nodulation-related genes in rhizobia) in B. yuanmingense was also measured (64) using a qPCR approach that was consistent with that mentioned above (Table S1).

**(iii) Rhizosphere microbial metabolic pattern analysis.** The microbial metabolic pattern of the rhizosphere soil (0- to 28-day soil samples collected from rhizosphere simulation experiment) was assessed using an Biolog EcoPlates system (Biolog, Inc., Hayward, CA, USA) (65, 66). Each 96-well Biolog EcoPlate consisted of three replicates comprising 31 sole carbon sources and a water blank. Briefly, the fresh soil samples (0.5 g) were shaken (300 rpm) with 49.5 ml of a 0.85% sterile NaCl solution with 5 g of glass beads (3-mm diameter) for 10 min at 25°C and then brought to a final dilution of 10^−3^. Subsequently, 150 μl of the clear supernatant was inoculated into each well of the Biolog EcoPlates. Inoculated plates were incubated in the dark at 25°C, and color development was read as absorbance every 24 h with an automated plate reader (MicroStation; Biolog, Hayward, CA, USA) at wavelengths of 590 nm and 750 nm. Thirty-one carbon sources were organized into six groups: carbohydrates (d-galactonic acid lactone, glucose-1-phosphate, methyl-d-glucoside, d-cellobiose, α-d-lactose, *i*-erythritol, d-xylose, d-mannitol, *N*-acetyl-d-glucosamine, dl-α-glycerol phosphate, d-galacturonic acid, and d-glucosaminic acid), amino acids (l-asparagine, l-arginine, l-Phenylalanine, l-serine, l-threonine, and glycyl-l-glutamic acid), carboxylic acids (γ-hydroxy butyric acid, itaconic acid, α-keto butyric acid, d-malic acid, and pyruvic acid methyl ester), amines (phenylethylamine and putrescine), phenolic acids (2-hydroxy benzoic acid and 4-hydroxy benzoic acid), and polymers (Tween 40, Tween 80, α-cyclodextrin, and glycogen). The overall rate of substrate utilization by microorganisms at 72 h of incubation was measured by calculating the average well color development (AWCD) as previously described (66). The relative utilization rate of each group of carbon sources was reflected by calculating the sum of the AWCDs of all carbon sources in the corresponding group.

### Field pot experiment 2 (synthetic root exudates improving peanut nodulation).

A synthetic root exudate experiment was performed to further test the proposed hypothesis that the specific root exudates caused by P. liquidambaris colonization could increase in nodulation and N_2_ fixation by improving PRFE. One kilogram of nonsterile soil was weighed and added to a plastic pot (15 cm in diameter and 13 cm high). The P. liquidambaris-colonized and noncolonized seedlings were transplanted into these pots (one seedling/pot), which were set as the P and CK treatments, respectively. In addition, specific root exudate components, such as organic acid, phenolics, and flavonoids, were added because their concentrations were significantly increased by P. liquidambaris colonization. Therefore, the synthetic root exudates (simulating the components derived by P. liquidambaris colonization) was composed of the following: organic acid (oxalic acid, 200 μg ml^−1^), phenolics (4-hydroxybenzoic acid, 0.25 μg ml^−1^; benzoic acid, 0.25 μg ml^−1^; coumaric acid, 0.25 μg ml^−1^; cinnamic acid, 0.25 μg ml^−1^), and flavonoids (daidzein, 0.05 μg ml^−1^; genistein, 0.05 μg ml^−1^; biochanin A, 0.05 μg ml^−1^). For the SRE treatment, 1 ml of synthetic root exudates was dropped into the noncolonized seedling root soil every day for 40 days. Other treatments received the equivalent amount of sterile distilled water. Experimental pots were randomly arranged in the Nanjing Normal University Botanical Garden, and watering was performed by hand.

The peanut plants were collected after 10 and 42 days of cultivation. The roots (10 days) were carefully cleaned with sterile distilled water, and the transcriptional activities of the symbiosis-related genes *SymRK* and *CCaMK* in the peanut-*Bradyrhizobium* interaction were determined. Moreover, the active nodules were excised from the roots (42 days) with scalpels to measure the nodule number and total nodule dry weight. Peanut growth parameters such shoot (leaf and stem) dry weight and root dry weight were also measured at 42 days of cultivation. The harvested shoot samples were ground to a fine powder, and the N concentration and fixed N_2_ were also measured. In addition, the rhizosphere soils of the peanuts were also collected to determine the concentrations of nitrate and ammonium and the abundance and diversity of AOA and diazotrophs communities.

### Statistical analyses.

All statistical analyses were performed using SPSS, version 13.0 (SPSS, Inc., Chicago, IL, USA). The normal distribution of data was checked using a Shapiro-Wilk’s test, and the homogeneity of variance was checked using Levene’s test. If necessary, the values were log transformed accordingly. When an analysis consisted of only a control and an experimental group, an independent *t* test was performed using SPSS, version 13.0, software. When three or more groups were compared, a one-way analysis of variance (ANOVA) was performed, followed by Tukey’s multiple-comparison test, with the comparisons between different treatments considered significantly different at a *P* value of *<*0.05. In addition, when the time component was considered in the experiments, the significance was calculated for the entire data set using repeated-measures ANOVA with Bonferroni’s *post hoc* test, and *P* value of *<*0.05 was considered significant. DGGE banding patterns were digitized and processed using Compare II software (Applied Maths, Austin, TX, USA). Cluster analysis of microbial proﬁles was performed using Gelcompar II to construct a dendrogram using the unweighted pair group method (UPGMA) based on the Pearson’s similarity coefficient calculated from the complete densitometric curves. The Shannon-Weaver index (*H*) was calculated from *H* = (*n_i_*/*N*) log(*n_i_*/*N*), where *n_i_* is the peak height of a band and *N* is the sum of all the peak heights in a lane. The DGGE fingerprint data were processed by generating a band-matching table, and the generated binary data were exported and compared by principal-component analysis (PCA) with CANOCO, version 4.5, software (Biometry, Wageningen, The Netherlands). Analysis of similarity (ANOSIM; Primer-E) was used to test for differences between categorical groups based on Bray-Curtis (67). ANOSIM was used to generate *R*, the correlation coefﬁcient, where an *R* value of 1 indicated complete separation between groups and a value of 0 indicated no separation. In addition, DGGE fingerprints were also analyzed by multivariate analyses by considering the environmental variables and the positions and intensities of individual bands in DGGE fingerprints species variables, using CANOCO, version 4.5, software. First, a detrended correlation analysis (DCA) was calculated, and a linear distribution was observed (length of gradient, >4), which showed that the best mathematical model to be applied to the data is the canonical correspondence analysis (CCA) (68). The significance of correlations between environmental and species variables was determined by inclusion of a Monte Carlo permutation test with 499 unrestricted permutations. Differences were considered significant at a *P* value of ≤0.05.

### Data availability.

The data supporting the ﬁndings of this study are available within the article and its supplemental material ﬁles or are available from the corresponding author upon request.
