# Supplementary material for: Endophytic Fungus Drives Nodulation and N2 Fixation Attributable to Specific Root Exudates
Source: mBio. 2019 Jul 16;10(4):e00728-19. doi: 10.1128/mBio.00728-19 (PMC6635524; doi:10.1128/mBio.00728-19)
Supplement: TABLE S2 [file mBio.00728-19-st002.docx]

**Table S2** Effects of root exudates on rhizosphere soil characteristics and nutrient concentrations

| Effects | | pH | TOC (g kg^-1^) | TN (g kg^-1^) | TP (g kg^-1^) | TK (g kg^-1^) | C:N |
| --- | --- | --- | --- | --- | --- | --- | --- |
|  |  |  |  |  |  |  |  |
| 0 d | | 5.27 ± 0.06 | 7.35 ± 0.13 | 0.65 ± 0.03 | 0.42 ± 0.02 | 11.51 ± 0.30 | 11.33 ± 0.46 |
| 3 d | H_2_O | 5.20 ± 0.10 | 7.22 ± 0.05^aA^ | 0.67 ± 0.03 | 0.45 ± 0.03 | 11.57 ± 0.24 | 10.79 ± 0.48 |
|  | CK | 5.30 ± 0.10 | 7.50 ± 0.07^abA^ | 0.65 ± 0.02 | 0.46 ± 0.04 | 11.61 ± 0.30 | 11.49 ± 0.44 |
|  | P | 5.27 ± 0.21 | 7.80 ± 0.13^bA^ | 0.65 ± 0.03 | 0.44 ± 0.02 | 11.58 ± 0.36 | 11.95 ± 0.42 |
| 7 d | H_2_O | 5.23 ± 0.06 | 7.29 ± 0.05^aAB^ | 0.67 ± 0.04 | 0.46 ± 0.03 | 11.67 ± 0.24 | 10.91 ± 0.66 |
|  | CK | 5.20 ± 0.10 | 7.79 ± 0.09^bA^ | 0.64 ± 0.03 | 0.44 ± 0.02 | 11.57 ± 0.31 | 12.12 ± 0.51 |
|  | P | 5.17 ± 0.06 | 8.23 ± 0.08^bB^ | 0.68 ± 0.03 | 0.46 ± 0.02 | 11.24 ± 0.31 | 12.18 ± 0.59 |
| 14 d | H_2_O | 5.30 ± 0.20 | 7.24 ± 0.09^aAB^ | 0.63 ± 0.03 | 0.45 ± 0.02 | 11.80 ± 0.33 | 11.56 ± 0.42 |
|  | CK | 5.20 ± 0.10 | 8.01 ± 0.08^bA^ | 0.64 ± 0.02 | 0.44 ± 0.05 | 11.64 ± 0.19 | 12.52 ± 0.43 |
|  | P | 5.17 ± 0.25 | 8.60 ± 0.14^cABC^ | 0.66 ± 0.04 | 0.48 ± 0.04 | 11.47 ± 0.26 | 12.99 ± 0.81 |
| 21 d | H_2_O | 5.13 ± 0.06 | 7.39 ± 0.07^aAB^ | 0.68 ± 0.02 | 0.46 ± 0.03 | 11.52 ± 0.30 | 10.87 ± 0.36^a^ |
|  | CK | 5.30 ± 0.10 | 8.04 ± 0.11^bA^ | 0.64 ± 0.03 | 0.47 ± 0.04 | 11.53 ± 0.31 | 12.51 ± 0.40^ab^ |
|  | P | 5.20 ± 0.10 | 8.69 ± 0.11^cABC^ | 0.68 ± 0.02 | 0.45 ± 0.03 | 11.43 ± 0.20 | 12.79 ± 0.44^b^ |
| 28 d | H_2_O | 5.23 ± 0.25 | 7.60 ± 0.07^aB^ | 0.65 ± 0.03 | 0.45 ± 0.03 | 11.51 ± 0.30 | 11.65 ± 0.46 |
|  | CK | 5.17 ± 0.12 | 8.14 ± 0.10^bA^ | 0.67 ± 0.02 | 0.45 ± 0.02 | 11.57 ± 0.25 | 12.21 ± 0.30 |
|  | P | 5.23 ± 0.06 | 8.79 ± 0.10^cC^ | 0.67 ± 0.02 | 0.44 ± 0.03 | 11.63 ± 0.22 | 13.20 ± 0.55 |

The values are the means ± SD from three biological replicates, with each biological replicate representing a pooled sample from at least five individual rhizosphere soil. For a column, different superscript lowercase letters indicate significant differences among different treatments at the same sampling times, and different superscript capital letters indicate significant differences among different sampling time at the same treatment. Same letters or no letters indicate no significant difference.

**Table S2** continued

| Effects | | C:P | N:P | DOC (mg kg^-1^) | DON (mg kg^-1^) | NH_4_^+^ (mg kg^-1^) | NO_3_^-^ (mg kg^-1^) |
| --- | --- | --- | --- | --- | --- | --- | --- |
|  |  |  |  |  |  |  |  |
| 0 d | | 17.53 ± 0.74 | 1.55 ± 0.00 | 42.56 ± 2.06 | 7.24 ± 0.11 | 2.37 ± 0.03 | 8.32 ± 0.07 |
| 3 d | H_2_O | 15.97 ± 0.98 | 1.48 ± 0.03 | 42.95 ± 1.71 | 7.24 ± 0.19^a^ | 2.35 ± 0.04^aBD^ | 8.30 ± 0.05^A^ |
|  | CK | 16.45 ± 0.77 | 1.43 ± 0.09 | 43.64 ± 1.90^A^ | 7.54 ± 0.21^aA^ | 2.44 ± 0.03^aA^ | 8.29 ± 0.13^A^ |
|  | P | 17.61 ± 0.79 | 1.47 ± 0.09 | 46.80 ± 1.89^A^ | 7.85 ± 0.19^bA^ | 2.46 ± 0.03^aA^ | 8.35 ± 0.09^A^ |
| 7 d | H_2_O | 16.01 ± 0.96 | 1.47 ± 0.16 | 42.60 ± 2.01 | 7.28 ± 0.16^a^ | 2.22 ± 0.03^aCD^ | 8.68 ± 0.06^aB^ |
|  | CK | 17.85 ± 0.70 | 1.48 ± 0.11 | 47.62 ± 0.81^AB^ | 7.82 ± 0.25^bA^ | 2.63 ± 0.03^bAB^ | 9.85 ± 0.12^cA^ |
|  | P | 17.80 ± 1.01 | 1.46 ± 0.08 | 48.50 ± 1.72^A^ | 8.56 ± 0.26^cB^ | 2.75 ± 0.03^cAB^ | 9.23 ± 0.10^bB^ |
| 14 d | H_2_O | 16.23 ± 0.89 | 1.41 ± 0.09 | 42.91 ± 2.28^a^ | 7.28 ± 0.25^a^ | 2.14 ± 0.03^aABC^ | 9.03 ± 0.10^aBCD^ |
|  | CK | 18.47 ± 1.88 | 1.48 ± 0.20 | 50.98 ± 1.40^aB^ | 8.11 ± 0.30^bA^ | 2.82 ± 0.03^bB^ | 11.34 ± 0.66^bAB^ |
|  | P | 18.11 ± 1.41 | 1.40 ± 0.18 | 56.25 ± 1.51^bA^ | 10.69 ± 0.50^cC^ | 3.15 ± 0.03^cBC^ | 9.84 ± 0.09^abBC^ |
| 21 d | H_2_O | 16.14 ± 1.41 | 1.48 ± 0.12 | 44.10 ± 1.11^a^ | 7.30 ± 0.31^a^ | 1.95 ± 0.03^aB^ | 9.37 ± 0.05^aBCD^ |
|  | CK | 17.17 ± 1.21 | 1.37 ± 0.13 | 57.44 ± 1.35^bC^ | 10.51 ± 0.57^aAB^ | 3.06 ± 0.04^bC^ | 12.67 ± 0.84^aAB^ |
|  | P | 19.23 ± 1.09 | 1.51 ± 0.13 | 66.68 ± 1.96^cB^ | 14.36 ± 0.76^bD^ | 3.42 ± 0.04^cCD^ | 10.74 ± 0.36^aABC^ |
| 28 d | H_2_O | 16.93 ± 1.02 | 1.45 ± 0.04 | 44.29 ± 1.70^a^ | 7.51 ± 0.31^a^ | 1.83 ± 0.03^aA^ | 9.83 ± 0.11^aD^ |
|  | CK | 18.23 ± 0.50 | 1.49 ± 0.07 | 59.39 ± 2.37^bC^ | 12.43 ± 0.69^bB^ | 3.25 ± 0.04^bD^ | 14.77 ± 0.52^cB^ |
|  | P | 19.91 ± 1.62 | 1.51 ± 0.07 | 73.46 ± 3.60^cB^ | 17.54 ± 0.91^cE^ | 3.88 ± 0.03^cD^ | 11.05 ± 0.21^bC^ |

**Table S2** continued

| Effects | | AP (mg kg^-1^) | AK (mg kg^-1^) | PNR (μg NO_2_^-^-N g^-1^ h^-1^) |
| --- | --- | --- | --- | --- |
|  |  |  |  |  |
| 0 d | | 12.37 ± 0.14 | 113.13 ± 4.51 | 0.23 ± 0.02 |
| 3 d | H_2_O | 12.50 ± 0.30^A^ | 112.11 ± 3.52 | 0.22 ± 0.02^A^ |
|  | CK | 12.74 ± 0.27^A^ | 118.69 ± 4.96 | 0.24 ± 0.02^AB^ |
|  | P | 12.98 ± 0.31^A^ | 113.99 ± 5.06^A^ | 0.24 ± 0.02^A^ |
| 7 d | H_2_O | 12.54 ± 0.26^aA^ | 115.16 ± 7.45 | 0.24 ± 0.03^A^ |
|  | CK | 13.14 ± 0.20^abAB^ | 122.84 ± 5.34 | 0.27 ± 0.03^A^ |
|  | P | 14.16 ± 0.24^bA^ | 129.24 ± 3.24^AB^ | 0.25 ± 0.03^A^ |
| 14 d | H_2_O | 12.89 ± 0.19^aAB^ | 118.61 ± 3.80^a^ | 0.28 ± 0.03^aA^ |
|  | CK | 13.90 ± 0.16^bA^ | 125.38 ± 6.51^ab^ | 0.35 ± 0.03^bB^ |
|  | P | 15.72 ± 0.17^cB^ | 139.37 ± 3.28^bB^ | 0.27 ± 0.03^aA^ |
| 21 d | H_2_O | 13.32 ± 0.48^aAB^ | 121.97 ± 4.45^a^ | 0.32 ± 0.02^aA^ |
|  | CK | 15.24 ± 0.20^bB^ | 136.27 ± 3.98^b^ | 0.43 ± 0.03^bC^ |
|  | P | 18.00 ± 0.40^cB^ | 147.90 ± 5.54^cBC^ | 0.32 ± 0.03^aA^ |
| 28 d | H_2_O | 13.65 ± 0.23^aB^ | 125.97 ± 6.12^a^ | 0.32 ± 0.03^aA^ |
|  | CK | 15.91 ± 0.29^bB^ | 142.98 ± 4.57^b^ | 0.56 ± 0.03^bD^ |
|  | P | 19.96 ± 0.33^cC^ | 153.24 ± 3.22^bC^ | 0.38 ± 0.03^aA^ |
