# Supplementary figures and images for: Endophytic Fungus Drives Nodulation and N2 Fixation Attributable to Specific Root Exudates
Source: mBio. 2019 Jul 16;10(4):e00728-19. doi: 10.1128/mBio.00728-19 (PMC6635524; doi:10.1128/mBio.00728-19)

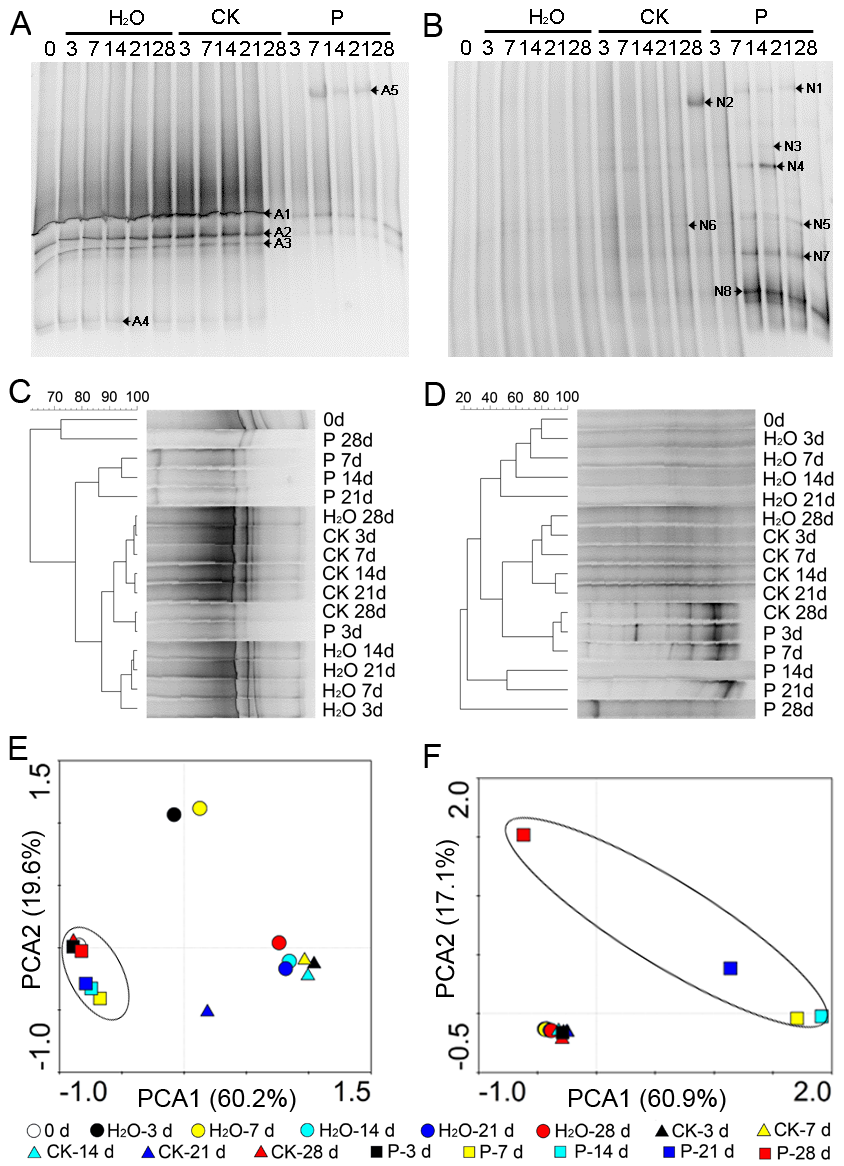

Supplement: FIG S1 [file mBio.00728-19-sf001.tif]

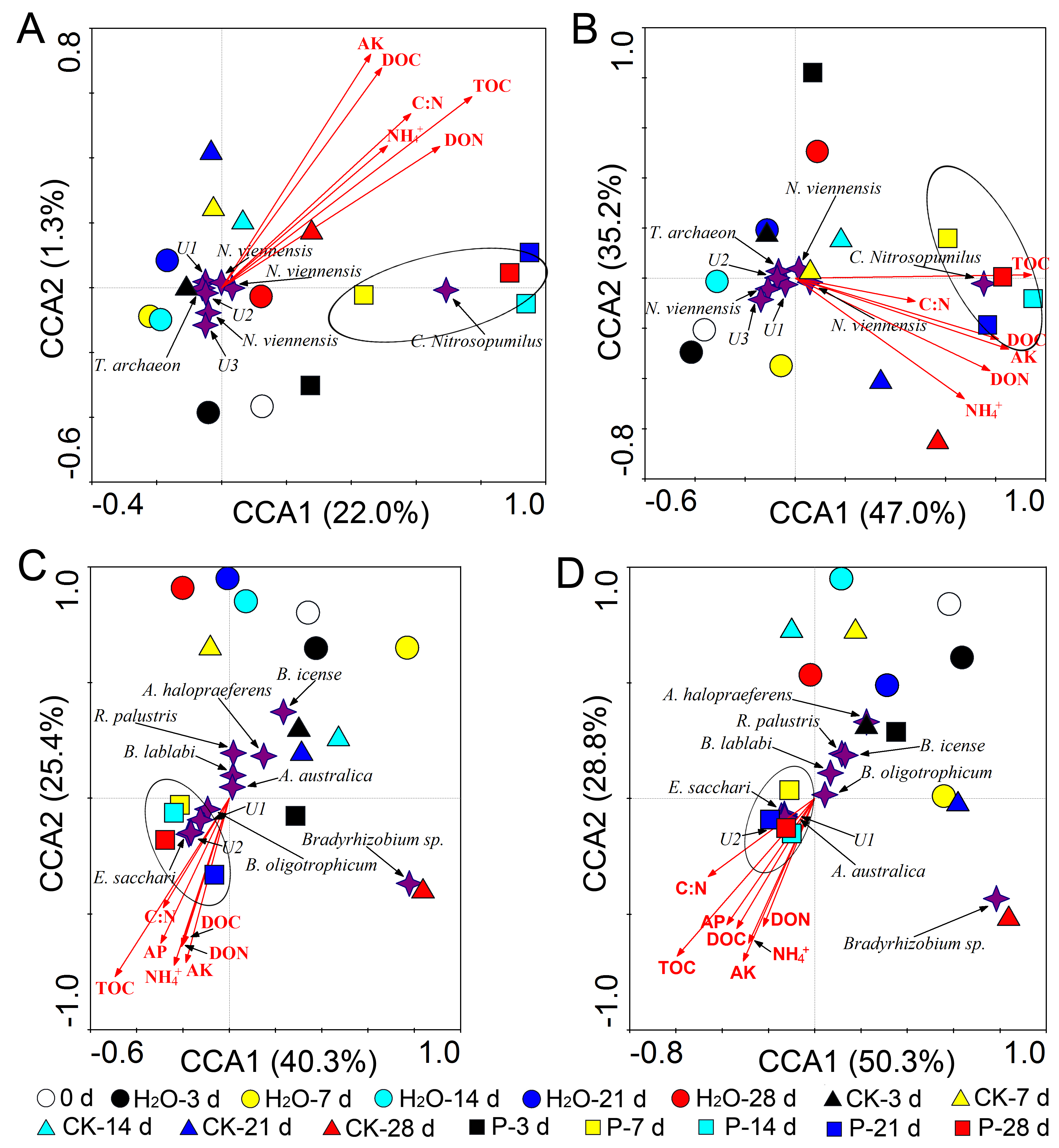

Supplement: FIG S2 [file mBio.00728-19-sf002.tif]

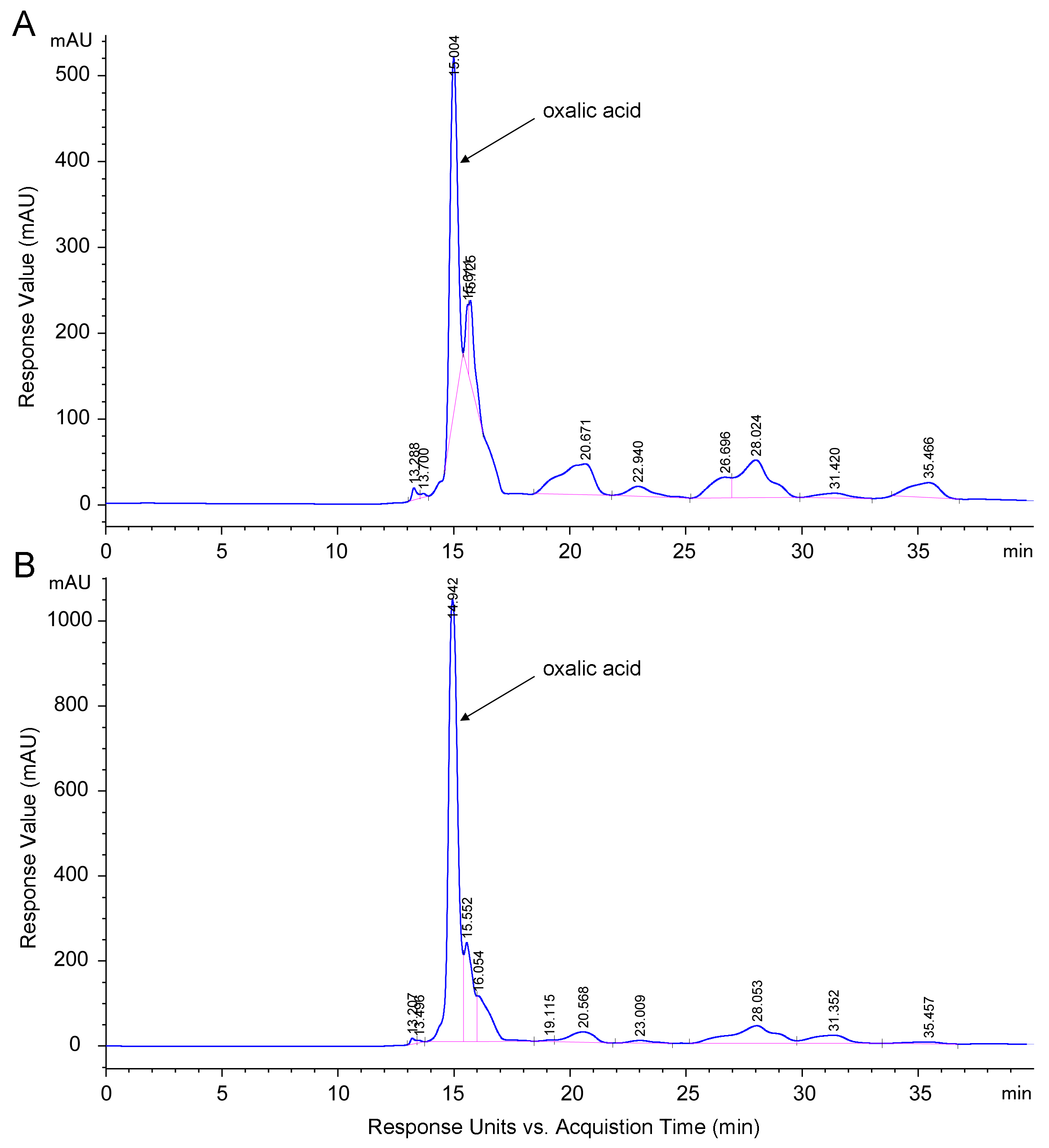

Supplement: FIG S3 [file mBio.00728-19-sf003.tif]

Fig. S4 (TIF file)


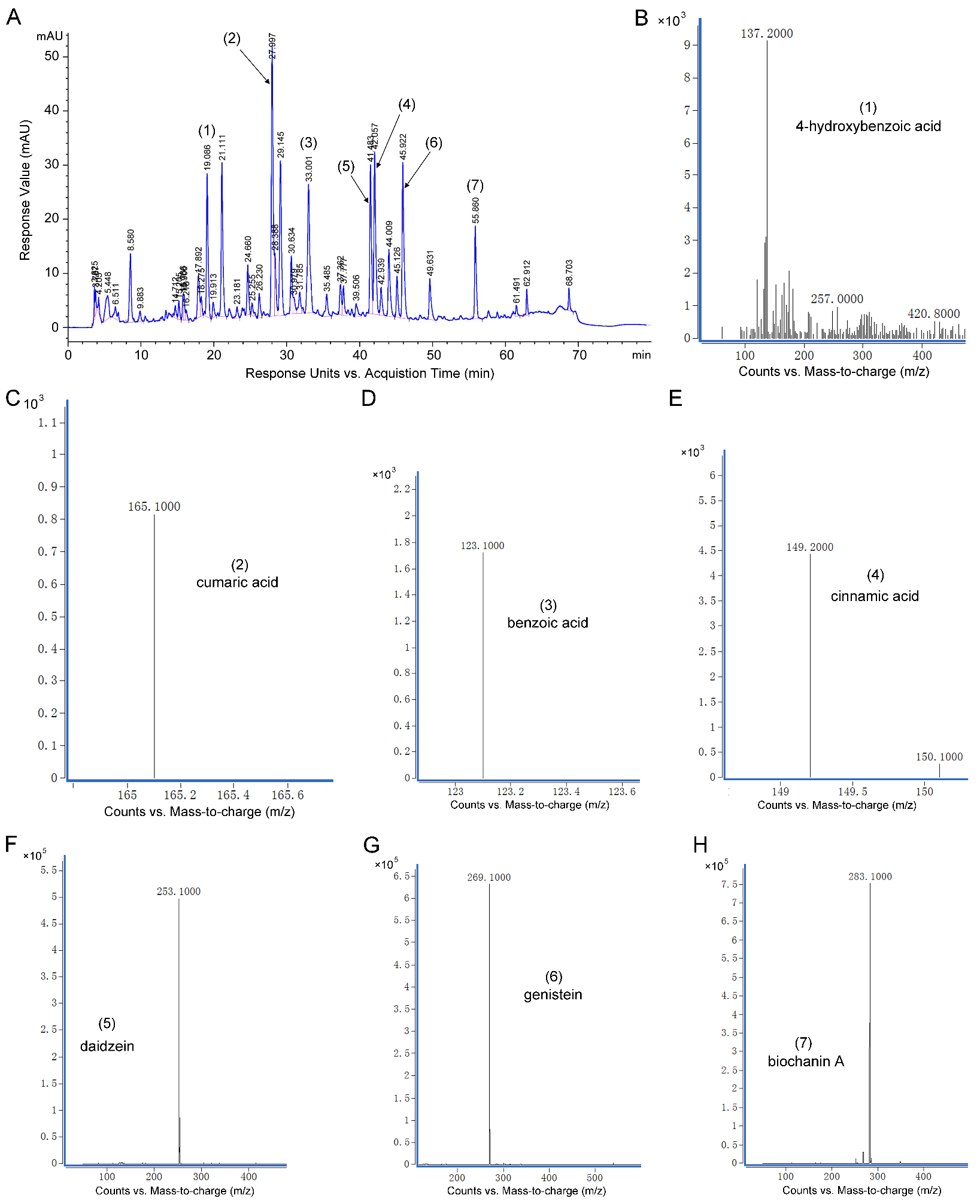

Supplement: FIG S4 [file mBio.00728-19-sf004.docx]
