# Supplementary material for: Endophytic Fungus Drives Nodulation and N2 Fixation Attributable to Specific Root Exudates
Source: mBio. 2019 Jul 16;10(4):e00728-19. doi: 10.1128/mBio.00728-19 (PMC6635524; doi:10.1128/mBio.00728-19)
Supplement: TABLE S3 [file mBio.00728-19-st003.docx]

**Table S3A** The DNA sequence identities of selected AOA and diazotroph DGGE bands in rhizosphere simulation experiments

| Band number | GenBank accession no. | Closest match from GenBank | Coverage/ID (100%) |
| --- | --- | --- | --- |
| A1^a^ | NZ_CP007536.1 | *Nitrososphaera viennensis* | 99/92 |
| A2 | NZ_CP007536.1 | *N. viennensis* | 99/91 |
| A3 | NZ_CBTY010000009.1 | *Thaumarchaeota archaeon* | 85/75 |
| A4 | NZ_CP007536.1 | *N. viennensis* | 99/79 |
| A5 | NC_018656.1 | *Candidatus Nitrosopumilus* sp. | 99/73 |
| N1^b^ | NZ_CP007215.2 | *Enterobacter sacchari* | 90/88 |
| N2 | NZ_CAFK01000249.1 | *Bradyrhizobium* sp. | 99/93 |
| N3 | NZ_KE386928.1 | *Azospirillum halopraeferens* | 100/91 |
| N4 | NZ_LLYB01000119.1 | *Bradyrhizobium lablabi* | 99/93 |
| N5 | NC_007778.1 | *Rhodopseudomonas palustris* | 99/90 |
| N6 | NZ_CP016428.1 | *Bradyrhizobium icense* | 99/93 |
| N7 | NZ_KE387211.1 | *Azohydromonas australica* | 97/90 |
| N8 | NC_020453.1 | *Bradyrhizobium oligotrophicum* | 99/94 |

“a” with the letter “A” represents clones obtained for the AOA DGGE proﬁles. “b” with the letter “N” represents clones obtained for the diazotroph DGGE proﬁles.

**Table S3B** The DNA sequence identities of selected AOA and diazotroph DGGE bands in synthetic root exudates experiments

| Band number | GenBank accession no. | Closest match from GenBank | Coverage/ID (100%) |
| --- | --- | --- | --- |
| A1^c^ | KM595439.1 | Uncultured *thaumarchaeota* | 100/99 |
| A2 | KM116942.1 | Uncultured *archaeon* | 99/99 |
| A3 | AB920297.1 | Uncultured *archaeon* | 100/99 |
| A4 | AB545943.1 | Uncultured *crenarchaeote* | 99/99 |
| N1^d^ | KR092338.1 | *Bradyrhizobium japonicum* | 100/100 |
| N2 | MF144116.1 | *Bradyrhizobium* sp. HY1 | 100/100 |
| N3 | KJ656136.1 | Uncultured *Bradyrhizobium* sp. | 100/99 |

“c” with the letter “A” represents clones obtained for the AOA DGGE proﬁles. “d” with the letter “N” represents clones obtained for the diazotroph DGGE proﬁles.
