# Supplementary material for: Endophytic Fungus Drives Nodulation and N2 Fixation Attributable to Specific Root Exudates
Source: mBio. 2019 Jul 16;10(4):e00728-19. doi: 10.1128/mBio.00728-19 (PMC6635524; doi:10.1128/mBio.00728-19)
Supplement: TABLE S4 [file mBio.00728-19-st004.docx]

**Table S4** Effects of root exudates on the richness and *Shannon*-*Weaver* indices of rhizosphere soil AOA and diazotroph communities by DGGE analysis

| Effects | | AOA | |  | Diazotroph | |
| --- | --- | --- | --- | --- | --- | --- |
|  |  | *S* | *H* |  | *S* | *H* |
| 0 d | | 7.00 ± 2.00 | 1.75 ± 0.19 |  | 2.33 ± 0.58 | 0.81 ± 0.21 |
| 3 d | H_2_O | 9.67 ± 1.53^b^ | 1.98 ± 0.12^b^ |  | 2.33 ± 0.58^a^ | 0.82 ± 0.22^a^ |
|  | CK | 8.67 ± 1.15^b^ | 1.79 ± 0.10^b^ |  | 6.00 ± 1.73^ab^ | 1.69 ± 0.30^ab^ |
|  | P | 3.00 ± 0.00^a^ | 0.87 ± 0.02^a^ |  | 8.00 ± 1.73^c^ | 2.01 ± 0.22^b^ |
| 7 d | H_2_O | 9.67 ± 1.53^b^ | 1.97 ± 0.11^b^ |  | 2.67 ± 0.58^a^ | 0.93 ± 0.12^a^ |
|  | CK | 8.33 ± 0.58^b^ | 1.78 ± 0.04^b^ |  | 6.00 ± 1.00^b^ | 1.73 ± 0.05^b^ |
|  | P | 3.67 ± 0.58^a^ | 1.14 ± 0.07^a^ |  | 10.67 ± 1.15^c^ | 1.96 ± 0.03^c^ |
| 14 d | H_2_O | 9.33 ± 0.58^b^ | 1.92 ± 0.06^b^ |  | 3.33 ± 1.15^a^ | 1.10 ± 0.06^a^ |
|  | CK | 7.67 ± 1.15^ab^ | 1.74 ± 0.10^ab^ |  | 6.67 ± 0.58^b^ | 1.82 ± 0.06^b^ |
|  | P | 3.33 ± 0.58^a^ | 1.11 ± 0.09^a^ |  | 10.33 ± 0.58^c^ | 2.00 ± 0.05^c^ |
| 21 d | H_2_O | 8.67 ± 1.15^b^ | 1.77 ± 0.04^b^ |  | 4.33 ± 2.52^a^ | 1.26 ± 0.08^a^ |
|  | CK | 7.33 ± 1.53^ab^ | 1.66 ± 0.13^b^ |  | 6.00 ± 1.00^ab^ | 1.71 ± 0.05^bc^ |
|  | P | 3.67 ± 0.58^a^ | 1.13 ± 0.08^a^ |  | 9.67 ± 0.58^b^ | 1.88 ± 0.05^c^ |
| 28 d | H_2_O | 9.00 ± 0.00^b^ | 1.86 ± 0.09^b^ |  | 4.00 ± 1.00^a^ | 1.30 ± 0.21^a^ |
|  | CK | 4.00 ± 1.00^a^ | 1.17 ± 0.17^ab^ |  | 7.00 ± 0.00^a^ | 1.42 ± 0.12^a^ |
|  | P | 4.00 ± 0.00^a^ | 1.08 ± 0.06^a^ |  | 8.00 ± 0.00^b^ | 1.53 ± 0.11^a^ |

The values are the means ± SD from three biological replicates, with each biological replicate representing a pooled sample from at least five individual rhizosphere soil. For a column, different superscript lowercase letters indicate significant differences among different treatments at the same sampling times, and different superscript capital letters indicate significant differences among different sampling time at the same treatment. Same letters or no letters indicate no significant difference.
