# Supplementary material for: Endophytic Fungus Drives Nodulation and N2 Fixation Attributable to Specific Root Exudates
Source: mBio. 2019 Jul 16;10(4):e00728-19. doi: 10.1128/mBio.00728-19 (PMC6635524; doi:10.1128/mBio.00728-19)
Supplement: TABLE S1 [file mBio.00728-19-st001.docx]

**Table S1** Primers used for the real-time PCR and PCR-DGGE analysis

| Target group | Name | Sequence (5'– 3') | References |
| --- | --- | --- | --- |
| *P. liquidambaris*-specific ITS gene | Bf1 | CTGGCCCCCTCGGGGTCCCTGG | Wang et al., 2014 |
|  | Br1 | TTTCAGGGCCTGCCCTTTTACAGGC |  |
| AOB (*amoA* gene) | GC^a^-amoA1F | GGGGTTTCTACTGGTGGT | Rotthauwe et al., 1997 |
|  | amoA2R | CCCCTCKGSAAAGCCTTCTTC |  |
| AOA (*amoA* gene) | Arch-amoAF | STAATGGTCTGGCTTAGACG | Francis et al., 2005 |
|  | GC^b^-Arch-amoAR | GCGGCCATCCATCTGTATGT |  |
| Diazotroph (*nifH* gene) | Pol F1 | TGCGAICCSAAIGCIGACTC | Wartiainen et al., 2008 |
|  | GC^c^-AQER | GACGATGTAGATYTCCTG |  |
| *PAL* (gene) | PAL-F | CGCTCTTAGAACTTCACCTC | This study |
|  | PAL-R | CAAGTGCTGGATCTGTGTTAT |  |
| *CHS* (gene) | CHS-F | AGGACTTGGCGGAGAAC | This study |
|  | CHS-R | ATCGGAACCAACAATGAGT |  |
| *CHI* (gene) | CHI-F | GACCCTGAAATAGTGAACC | This study |
|  | CHI-R | CCATACTGTGCTCCCTT |  |
| *nodC* (gene) | noC-F | TGATYGAYATGGARTAYTGGCT | Sarita et al., 2005 |
|  | noC-R | CGYGACARCCARTCGCTRTTG |  |

S = C/G; K = G/T; Y= C/T; R = A/G; Modiﬁed bases; I = Inosine

^a^ (CGCCCGCCGCGCCCCGCGCCCGTCCCGCCGCCCCCGCCCG)

^b^ (CGCCCGCCGCGCCCCGCGCCCGGCCCGCCGCCCCCGCCCC)

^c^ (CGCCCGCCGCGCCCCGCGCCCGGCCCGCCC)
